# ﻿Review of the *Messorsemirufus* complex (Hymenoptera, Formicidae) in Greece

**DOI:** 10.3897/zookeys.1185.111484

**Published:** 2023-11-28

**Authors:** Sebastian Salata, Albena Lapeva-Gjonova, Christos Georgiadis, Lech Borowiec

**Affiliations:** 1 University of Wrocław, Department of Biodiversity and Evolutionary Taxonomy, Myrmecological Laboratory, Przybyszewskiego 65, 51-148 Wrocław, Poland; 2 Department of Zoology and Anthropology, Faculty of Biology, Sofia University, 1164 Sofia, Bulgaria; 3 Section of Zoology and Marine Biology, Department of Biology, National and Kapodistrian University of Athens, 15784 Athens, Greece; 4 Museum of Zoology, Department of Biology, National and Kapodistrian University of Athens, 15772 Athens, Greece

**Keywords:** Balkans, Bulgaria, eastern Mediterranean, identification key, Myrmicinae, new species, redescription

## Abstract

*Messor* is a diverse genus of Myrmicinae with 168 extant species and subspecies. In the Mediterranean, some of its taxa historically were classified as members of the *Messorinstabilis* group (sensu Santschi), of which 19 are known from the eastern Mediterranean. Here, the *Messorsemirufus* complex of the Balkan Peninsula that assembles a distinct subsection of members of the *instabilis* group is defined and treated. In total, five species are recorded, including three that are new. *Messoratanassovii* Atanassov, 1982 is redescribed and confirmed for Bulgaria (Thracian Plain, Struma, and Mesta Valley, Pirin Mt., and Eastern Rhodopi) and Greece (Epirus, Ionian Islands, Central and Eastern Macedonia, and Thraki). Three species are described as new to science: *Messordanaes* Salata, Georgiadis & Borowiec, **sp. nov.** (Cyclades: Serifos), *Messorkardamenae* Salata & Borowiec, **sp. nov.** (Dodecanese: Kos, Nisyros, Rhodes, and Tilos), and *Messorveneris* Salata, Georgiadis & Borowiec, **sp. nov.** (Cyclades: Milos). The fifth member of the complex, *Messorcreticus* Borowiec & Salata, 2019, maintains its status of Cretan endemic.

## ﻿Introduction

*Messor* Forel, 1890 is a moderately large genus counting 168 species and subspecies and a single fossil species (Bolton 2023) distributed in the Palearctic, Afrotropical, and Oriental regions. The species of this genus are mainly granivorous and play a significant role in seed dispersal (De Almeida et al. 2020). Most of the *Messor* taxa are known from open and arid habitats, such as savannahs, grasslands, phrygana, semideserts, and deserts. However, there are some reports of *Messor* from steppes and open sites placed at high altitudes in the Mediterranean basin (Bolton 1982; Salata and Borowiec 2019).

Approximately 90 taxa of this genus are known from the Mediterranean subregion (Borowiec 2014). Still, the number is most likely underestimated as several species complexes have unclear status and require comprehensive revision that could reveal several cryptic or subcryptic taxa, as proved by Steiner et al. (2018) in their most recent revision of the *Messorstructor* species group. Moreover, many of the Mediterranean *Messor* taxa were proposed as intraspecific names of various ranks, partly available to nomenclature, which impedes certain estimations of the diversity of this genus in this region. Additionally, due to the complex taxonomic history of most of the Eastern Mediterranean *Messor* species combined with scarce material and ambiguous descriptions, their historical distribution records are problematic and preclude mapping their distribution ranges.

Workers of the genus *Messor* are polymorphic and very often the shape of the head, its sculpture cover and intensity, as well as the shape of the propodeal convexity are strongly correlated with the size of the specimen. Minor workers usually have weaker sculpture, in some cases the sculpture is absent, and their propodeum is rounded and lacks projections or denticles. Contrastingly, major workers of the same species can bear very dense and robust sculpture and their propodeum has distinct projections or denticles (Steiner et al. 2018). With this pattern of size-dependent morphological variation, this taxon is considered one of the most taxonomically challenging genera.

So far within the Mediterranean Region, only members of the *Messorstructor* group have been revised recently (Steiner et al. 2018). On a narrower scale, some preliminary contributions to studies on Greek *Messor* were presented by Salata and Borowiec (2019), who reported 11 species for this country. Three of the Greek *Messor* are endemic: *M.concolor* Santschi, 1927 and *M.creticus* Salata & Borowiec, 2019 are known only from Crete, and *M.carpathous* Menozzi, 1936 was described from Karpathos.

The *Messorsemirufus* complex belongs to the *Messorinstabilis* species group sensu Santschi (1927). This complex is characterised by small to medium body size with mean head width (HW) of the largest workers less than 2.7 mm (often less than 2.0 mm), small eyes, uniformly black or black and red body coloration, reduced head sculpture (lack of head striation or with striae present only on the frons), presence of psammophore on the ventral side of the head, propodeum lacking denticles, and first gastral tergite lacking erect setae or with only very scarce, extremely short erected setae placed on its basal part and occasionally also few additional setae placed close to its posterior margin. The remaining members of the *Messorinstabilis* species group known from the Eastern Mediterranean Region (grouped as *Messorwasmanni* complex here) differ in having a larger and usually bicoloured body (HW > 2.8 mm) and by the presence of at least one of the following characters: head sculpture more developed with striae covering at maximum the whole frontal head, lack of psammophore on the ventral side of the head, propodeum with denticles, and first gastral tergite with numerous erect setae.

In this paper, we reviewed the *Messorsemirufus* species complex, which includes five species from Greece, including three new to science. We also provided a synopsis of species of the *Messorinstabilis* species group known from the Eastern Mediterranean Region and a key to all Greek taxa belonging to this group. The number of *Messor* species known from Greece has increased to 15.

## ﻿Materials and methods

Examined specimens are housed in the following collections:

**BFUS**Faculty of Biology, Sofia University, Bulgaria;

**MHNG**Museum d’Historie Naturelle, Geneve, Switzerland;

**MNHW** Museum of Natural History, University of Wrocław, Poland;

**NMNH**National Museum of Natural History, Sofia, Bulgaria;

**USMB**Upper Silesian Museum, Bytom, Poland.

Specimens were compared using standard methods of comparative morphology. All measurements were made in mm using a pin-holding stage, permitting rotations around the X, Y, and Z axes. Photographs were taken using a Nikon SMZ 1500 stereomicroscope, Nikon D5200 camera, and Helicon Focus software. All given label data of type specimens are in the original spelling, and a vertical bar (|) separates data on different rows, and double vertical bars (||) separate labels. Images of type specimens are available online on AntWeb (www.AntWeb.org) and are accessible using the unique CASENT identifying specimen code.

The pilosity inclination degree applies to this used in Wilson (1995). The adpressed (0–5°) hairs run parallel, or nearly parallel to the body surface. Decumbent hairs stand 10–15°, subdecumbent hair stand 30°, suberect hairs stand 35–45°, and the erect hairs stand more than 45° from the body surface. The term “large setae” refers to setae that are longer than half of the length of the maximum vertical diameter of the eye of the investigated specimen. The descriptions of minor workers mention only these characters that differ compared to major workers.

### ﻿Measurements and abbreviations used

**EL** eye length; measured along the maximum vertical diameter of eye;

**HL** head length; measured in straight line from mid-point of anterior clypeal margin to mid-point of posterior margin in full-face view;

**HW** head width; measured in full-face view directly above the eyes;

**MW** mesosoma width; maximum width of promesonotum;

**PPW** postpetiole width; maximum width of postpetiole in dorsal view;

**PI** petiole index, length/width of petiole ratio;

**PSL** propodeal spine length; because species of the *Messorsemirufus* complex have no propodeal spines it is measured from the centre of the propodeal spiracle to the top of the propodeal angulation in lateral view;

**PW** petiole width; maximum width of petiole in dorsal view;

**SL** scape length; maximum straight-line length of scape excluding the articular condyle;

**WL** mesosoma length; measured as diagonal length from the anterior end of the neck margin to the posterior margin of the propodeal lobe;

**w.** worker;

**g.** gyne.

The Greek material was compared with the type material and fresh specimens of the following species of the *Messorsemirufus* complex known from the Mediterranean basin:

*Messorbouvieri* Bondroit, 1918: 4w. from Portugal (MNHW), 21w. from Spain (MNHW).

*Messorebeninus* Santschi, 1927: syntype of major worker: “M.barbarusL.r.semirufus And. v. *ebenina* Forel, Type, Doummar, Syrie” [type images examined, AntWeb, CASENT0901472, photos by Z. Lieberman, available on https://www.AntWeb.org]; syntype of minor worker: “M.barbarusL.r.semirufus And. v. *ebenina* Forel, Type, Djebel Kasioun Antiliban (Kerville)” [type images examined, AntWeb, CASENT0907735, photos by Alexandra Westrich, available on https://www.AntWeb.org] + 26w. from Iran (MNHW).

*Messorintermedius* Santschi, 1927: syntypes of major and minor worker: “M.barbarusssp.semirufus And. v. *intermedia* Forel, Type, Doummar, Syrie (Kerville) 22” [type images examined, AntWeb, CASENT0907736 and CASENT0907737, photos by Alexandra Westrich, available on https://www.AntWeb.org] + 1w. from Syria (MNHW) and 33w. from Iran (MNHW).

*Messormaculifrons* Santschi, 1927: 5w. from Israel (MNHW).

*Messorminor* (André, 1883): 14w. from Italy (MNHW).

*Messorminor* spp. *calabricus* Santschi, 1927: 2w. from Italy (MNHW).

*Messorsemirufus* (André, 1883): syntype of major worker: “*M.barbarus* v *semirufus* Andre, Cotypus, Syria, 1899 Morice” [type images examined, AntWeb, CASENT0907730, photos by Alexandra Westrich, available on https://www.AntWeb.org]; 5w. from Israel (MNHW) and 6w from Jordan (MNHW).

*Messorsyriacus* Tohmé, 1969; syntype of major worker: “*M.aralocaspiuslaboriosus* v. *syriacus* Sants., Type, Syrie, Damas., (G. de Kerville)” [type images examined, AntWeb, CASENT0913178, photos by Alexandra Westrich, available on https://www.AntWeb.org] + 26w. from Cyprus (MNHW) and 86w. from Iran (MNHW).

## ﻿Results

### ﻿Synopsis of members of the *Messorinstabilis* species group from the Eastern Mediterranean Region

I. *Messorwasmanni* complex.

1. *Messorcaducus* (Motschoulsky, 1839) (Armenia, Georgia, Iran, Kazakhstan, Turkey).

2. ^*^Messorconcolor Santschi, 1927 (Greece).

3. *Messordentatus* Santschi, 1927 (the Middle East).

4. *Messorhebraeus* Santschi, 1927 (Israel and Palestine, Lebanon, Syria).

5. *Messormeridionalis* (André, 1883) (uncertain).

6. *Messormediosanguineus* Donisthorpe, 1946 (Turkey).

7. *Messorsultanus* Santschi, 1917 (the Middle East).

8. ^*^Messorwasmanni Krausse, 1910 (the Balkans, western Turkey).

II. *Messorsemirufus* complex.

1. ^*^Messoratanassovii Atanassov, 1982: 209 (Bulgaria and Greece).

2. ^*^Messorcreticus Salata & Borowiec, 2019 (Greece: Crete).

3. ^*^Messordanaes Salata, Georgiadis & Borowiec, sp. nov. (Greece: Cyclades).

4. *Messorebeninus* Santschi, 1927: 229 (the Middle East, eastern Turkey).

5. *Messorintermedius* Santschi, 1927: 229 (the Middle East).

6. ^*^Messorkardamenae Salata & Borowiec, sp. nov. (Greece: Dodecanese).

7. *Messormaculifrons* Santschi, 1927: 228 (Egypt, Israel, Syria).

8. *Messorsemirufus* (André, 1883): 355 (Armenia, eastern Turkey, the Middle East).

9. *Messorsemirufusemeryi* Santschi, 1927 (Israel and Palestine, Lebanon).

10. *Messorsyriacus* Tohmé, 1969 (Cyprus, eastern Turkey, the Middle East).

11. ^*^Messorveneris Salata, Georgiadis & Borowiec, sp. nov. (Greece: Cyclades).

### ﻿Key to the species of the *Messorinstabilis* group from Greece

Note: Proper identification is possible only for a series of major workers.

**Table:** 

1	Large species, mean HW in the largest workers > 2.6 mm	**2**
–	Smaller species, mean HW in the largest workers < 2.5 mm	**4**
2	Occipital corners with 1–4 (only occasionally 5 or 6) large setae. Postpetiole with narrowing attachment to the gaster, PPW/PW index in major workers < 1.3 (mean 1.21)	**3**
–	Occipital corners with 7–11 (occasionally 12 or 13) large setae. Postpetiole with broad attachment to the gaster, PPW/PW index in major workers > 1.3 (mean 1.39). Only Dodecanese	***M.kardamenae* Salata & Borowiec, sp. nov.**
3	First gastral tergite with strong background microreticulation; pits around bases of setae in basal part of gaster form short and deep longitudinal striae. Mesosoma in large workers often bicoloured, red with black patches, in the largest workers often almost entirely black. Crete	***M.concolor* Santschi**
–	First gastral tergite with moderate background microreticulation, often reduced; pits around bases of setae in basal part of gaster **either** do not form longitudinal striae **or** form only short and shallow striae. Mesosoma in large workers usually completely red or with small brown to black patches, never entirely black. Widespread in the Balkans but in Crete mostly in coastal areas or in tourist resorts	***M.wasmanni* Krausse**
4	Mesosoma completely to predominantly red or predominantly brown to black with reddish discoloration on sides of mesonotum and dorsum of propodeum (Figs 1–9, 29–32), if mesosoma completely black then occipital part of head with 12–20 large setae	**5**
–	Mesosoma completely black, only occasionally anterolateral corners of pronotum with reddish discoloration (Figs 15–18). Occipital part of head always with less than 12 large setae. Cyclades: Serifos	***M.danaes* Salata, Georgiadis & Borowiec, sp. nov.**
5	Occipital area and vertex of head with 4–8 large setae (Figs 29, 31, 33)	**6**
–	Occipital area and vertex of head with 12–20 large setae (Figs 1, 3, 10)	***M.atanassovii* Atanassov**
6	Pronotum with thin and dense sculpture, its dorsum with reduced sculpture and sometimes with smooth patches centrally; first gastral tergite never with erect setae (Figs 21, 23). Milos Island of Cyclades	***M.veneris* Salata, Georgiadis & Borowiec, sp. nov.**
–	Propodeum with thick and sparser sculpture, its dorsum never with reduced sculpture or smooth patches; gaster with sparse and erect setae. Endemic to Crete	***M.creticus* Salata & Borowiec**

### ﻿Review of species

#### ﻿ Messor atanassovii

Taxon classificationAnimaliaHymenopteraFormicidae

Atanassov, 1982

3DDE2BDE-CF68-5D9D-8D52-10DD327A7B94

Figs 1–2 , 3, 4 , 5, 6 , 7–9 , 10, 11 , 12, 13 , 14

Messor atanassovii Atanassov, 1982: 209.
Messor cf. ebeninus : Bračko et al. 2018: 22.
Messor cf. semirufus : Bračko et al. 2018: 23.

##### Comments.

The location of the type material is unknown. AL-G unsuccessfully searched for the type specimens of *Messoratanassovii* in the collection of the Institute of Zoology of the Academy of Sciences in Sofia, which is indicated as the type material depository in the original description. However, during the search, it was revealed that currently the collection of Neno Atanassov is stored at the National Museum of Natural History, Sofia, Bulgaria. The investigation of the material revealed eight worker specimens of *Messor* with a label with a registration number “2183”, which corresponds to the number assigned to the part of paratypes mentioned in Atanassov (1982). Although the morphological characteristics of the specimens match those mentioned in the species description, there are no collecting, identification, or designation labels attached to any of them. Thus, AL-G organised an expedition to the sites indicated as terra typica of the species at the site near Belozem Village (east of Plovdiv) and collected a nest sample with *Messor* specimens bearing all the characters given in the original description of *Messoratanassovii* (topotypes).

##### Material examined.

Bulgaria • 8w. (pin) (NMNH); 10w. (pin), 17w. (EtOH); Thracian Plain, Belozem, Kisimovi dupki; 42.19002, 25.0291; 143 m; 06 May 2022; leg. A. Lapeva-Gjonova. • 6w. (pin), 1 g. (pin); same place; 21 Aug 2022; leg. A. Lapeva-Gjonova. • 8w. (pin), 20w. (EtOH); Thracian Plain, Dalbok izvor; 42.02179, 25.0908; 214 m; 08 May 2022; leg. A. Lapeva-Gjonova. • 3w. (pin), 15w. (EtOH); Mesta Valley, Petrlik; 41.49492, 23.86463; 484 m; 22 Jun 2022; leg. D. Gradinarov. • 3w. (pin), 15w. (EtOH); Eastern Rhodopes, Piyavets; 41.48776, 25.46150; 578 m; 26 Aug 2022; leg. A. Lapeva-Gjonova. • 3w. (pin), 12w. (EtOH); Pirin Mt., Lozenitsa; 41.50545, 23.35295; 288 m; 20 Nov 2022; leg. A. Lapeva-Gjonova. • 6w. (pin); Struma Valley, Kresna; 41.76534, 23.15566; 211 m; 06 May 2023; leg. I. Gjonov. GREECE• 4w. (pin); Central Macedonia, Pieria, Nei Pori; 39.974, 22.662; 1 m; 15 Jun 2013; leg. W. Żyła. • 2w. (pin); Eastern Macedonia and Thraki, Arriana, 2.5 km W of Kampos; 41.18714, 24.84118; 840 m; 30 Apr 2014; leg. G. Bračko. • 2w. (pin): Eastern Macedonia and Thraki, Xanthi, 1.5 km N of Kirnos; 41.0013, 24.77465; 20 m; 27 Apr 2014; leg. G. Bračko. • 3w. (pin); Eastern Macedonia and Thraki, Xanthi, Stavroupoli, 2 km NW of Dafonas; 41.22799, 24.6605; 220 m; 28 Apr 2014; leg. G. Bračko. • 35w. (pin); Epirus, Ammoudia; 39.2446, 20.48102; 1 m; 07 Sep 2016; leg. L. Borowiec. • 2w. (pin); Ionian Islands, Cephalonia, Skala; 38.07823, 20.79594; 38 m; 06 Jun 2019; leg. L. Borowiec. • 1g., 22w. (23 pin, 4 EtOH); Ionian Islands, Lefkada, 2.5 km S of Egklouvi; 38.708763, 20.63711; 1010 m; 02 Sep 2016; leg. L. Borowiec.

**Figures 1, 2.**
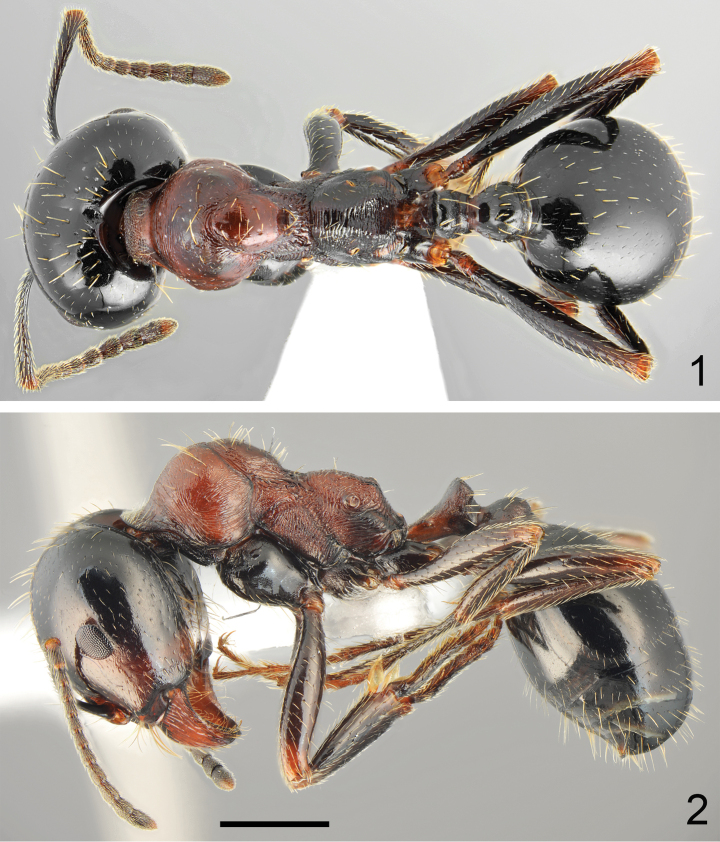
Major worker of *Messoratanassovii* Atanassov, 1982, population from Epirus **1** dorsal **2** lateral. Scale bar: 1 mm.

##### Description.

***Measurements*.** Major workers (*n* = 5): HL: 1.820–2.133 (mean 1.903); HW: 1.860–2.300 (mean 1.980); SL: 1.380–1.560 (mean 1.424); EL: 0.325–0.414 (mean 0.350); WL: 2.317–2.733 (mean 2.395); MW: 1.079–1.341 (mean 1.150); PSL: 0.238–0.317 (mean 0.278); PW: 0.381–0.476 (mean 0.409); PPW: 0.489–0.569 (mean 0.544); HL/HW: 0.927–0.984 (mean 0.962); SL/HW: 0.678–0.742 (mean 0.721); WL/MW: 2.038–2.260 (mean 2.132); EL/HL: 0.178–0.194 (mean 0.184); PSL/HW: 0.128–0.151 (mean 0.140); PPW/PW: 1.283–1.384 (mean 1.328).

**Figures 3, 4.**
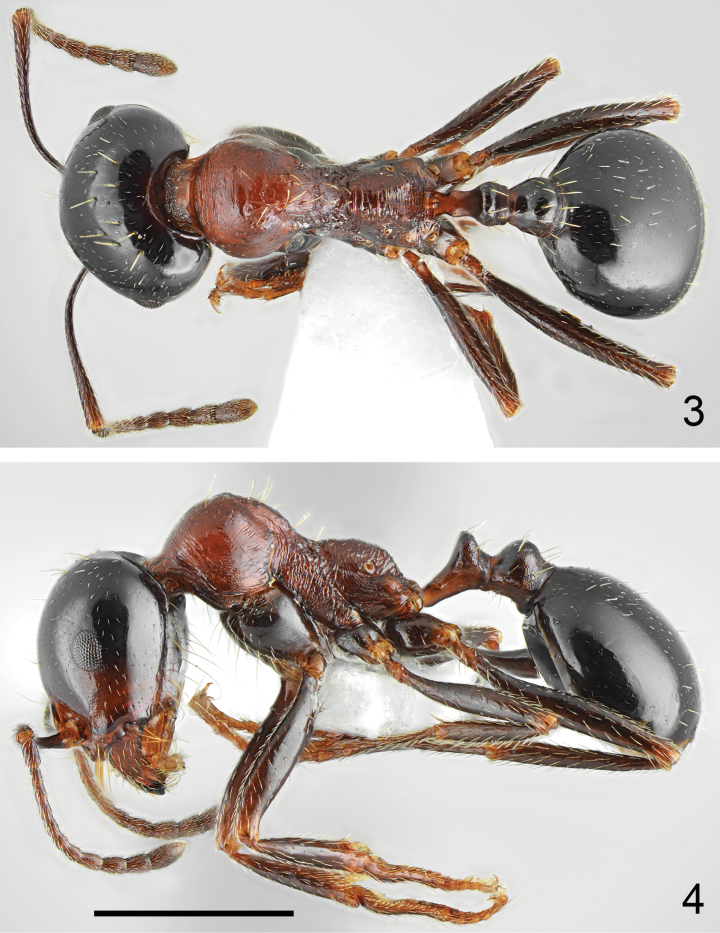
Minor worker of *Messoratanassovii* Atanassov, 1982, population from Epirus **3** dorsal **4** lateral. Scale bar: 1 mm.

**Figures 5, 6.**
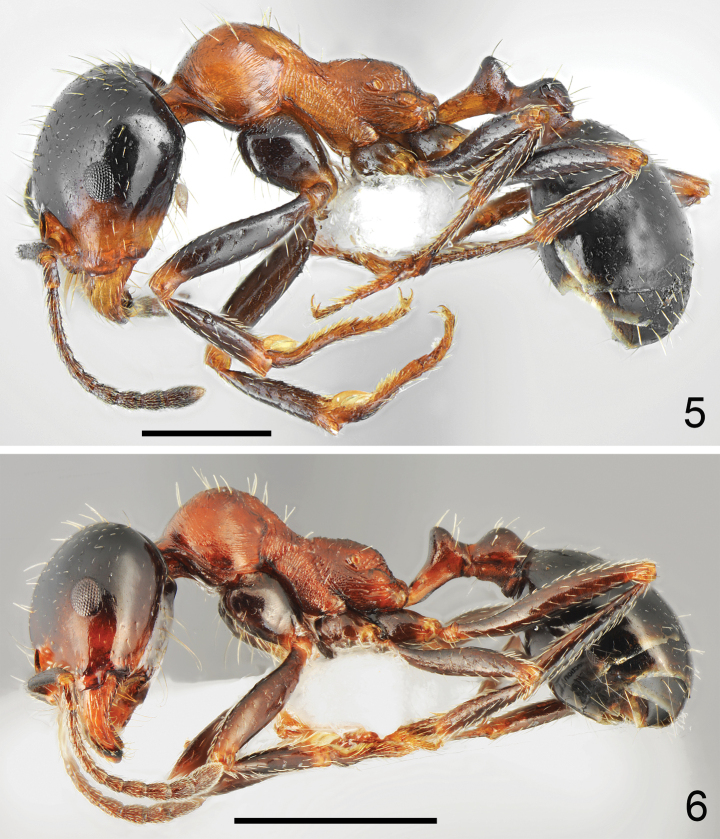
The palest specimens of *Messoratanassovii* Atanassov, 1982, population from Epirus **5** medium worker **6** minor worker. Scale bar: 1 mm.

**Figures 7–9.**
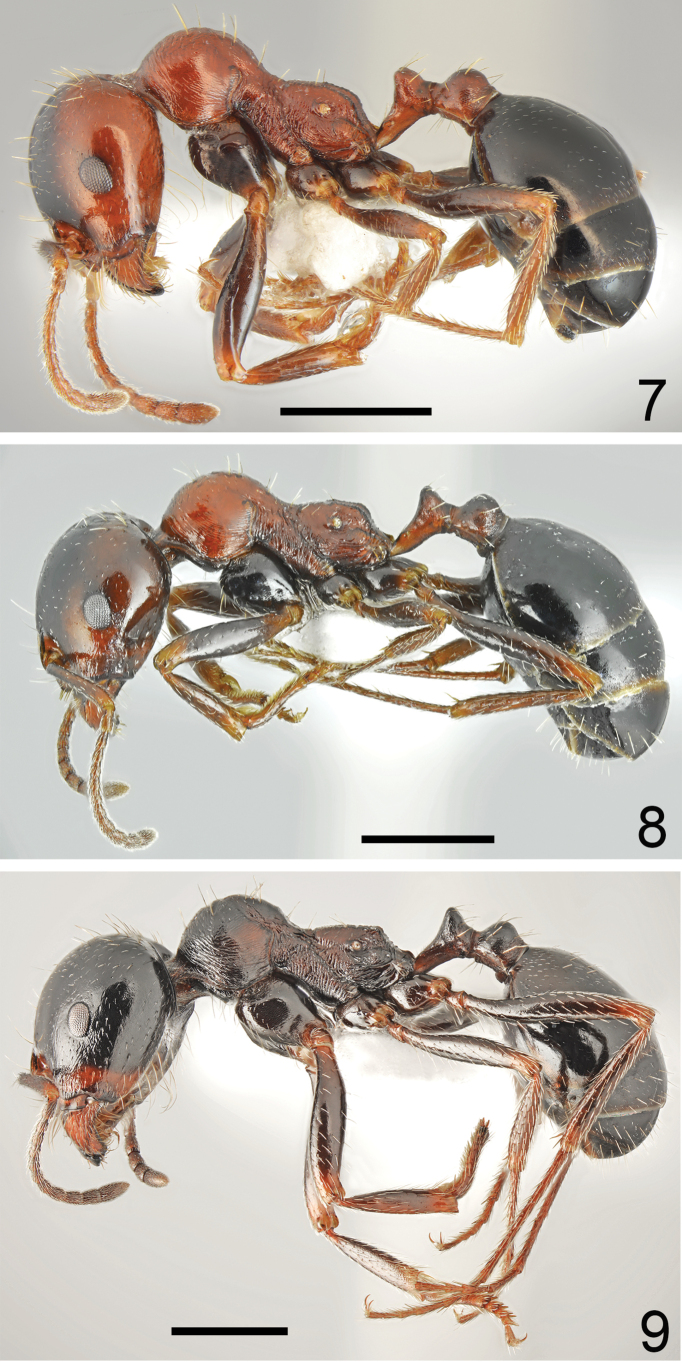
Workers of *Messoratanassovii* Atanassov, 1982 of population from Thraki **7, 8** medium workers **9** major worker. Scale bar: 1 mm.

**Figures 10, 11.**
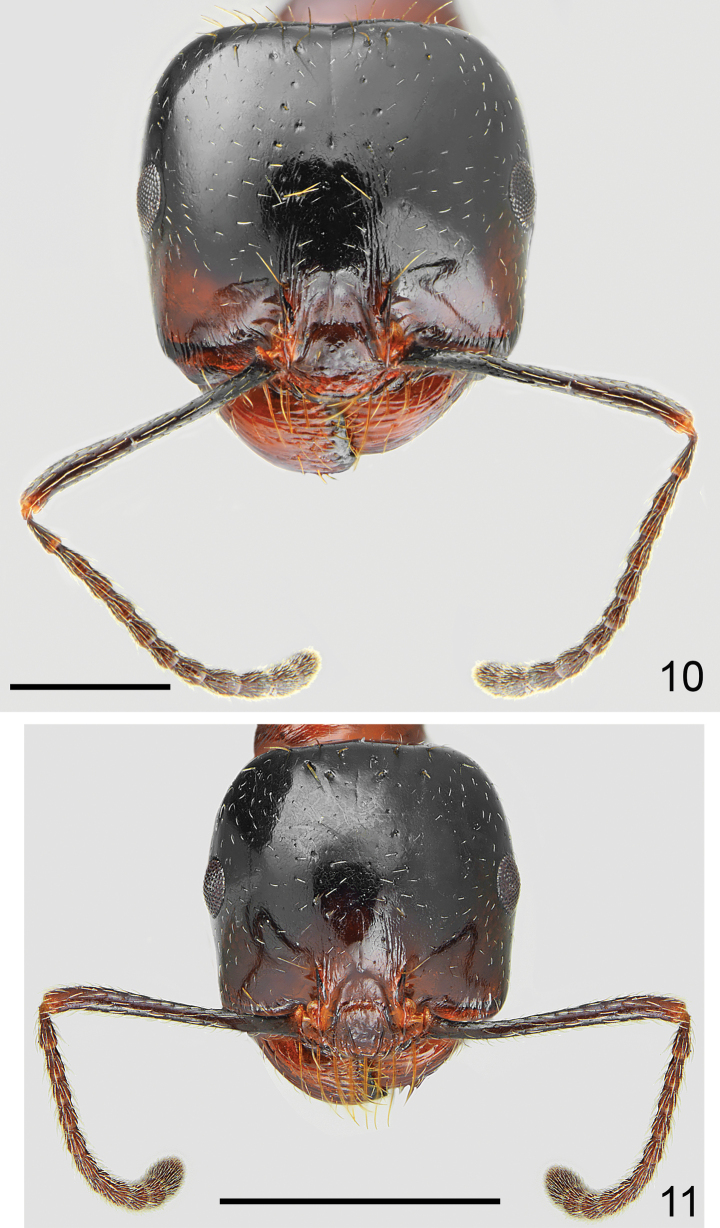
Head of *Messoratanassovii* Atanassov, 1982, population from Epirus **10** major worker **11** minor worker. Scale bar: 1 mm.

***Colour*.** Variable. Head mostly black, only gena, mandibles and frontal triangle reddish (Figs 1, 2, 5). In pale specimens, frons brown to black, and rest of head reddish to reddish brown (Figs 7, 8). Mesosoma in most specimens completely or predominantly red (Figs 1–8), in dark specimens mesosoma predominantly brown to black with reddish to brown patches (Fig. 9), occasionally whole mesosoma black with indistinct brown patch on mesonotum. Petiole and postpetiole reddish (Fig. 6), or only with base reddish and nodes brown to black (Figs 3–5). Gaster black, sometimes with reddish or yellowish transparent hind margins. Coxa reddish brown to brownish black, femora and tibiae brown to black with yellowish to reddish knee, basal segments of hind and mid tarsi brown, apical segments of hind and mid tarsi, and whole fore tarsi yellowish to reddish. Antennal scapes mostly dark brown to black with yellowish apex, funicle usually completely brown or dark brown basally and yellowish brown apically (Figs 10, 11). ***Head*.** Subrectangular, 0.93–0.98× as long as wide, sides below eyes subparallel, above eyes subparallel then softly rounded, posterior margin shallowly concave (Fig. 10). Anterior clypeal margin straight, without median emargination, with a row of 6–8 long marginal, yellowish setae, the longest as long as length of clypeus. Clypeus without appressed pubescence, laterally with a single erect seta and long erect seta close to the middle of lateral margin of central plate. Clypeus centrally with variable sculpture, from irregular rugosities to mostly smooth, without or with few longitudinal rugae, without median keel, interspaces microreticulate but shiny (Fig. 10). Eyes small and broadly oval, 0.5–0.6× as long as the gena. Frontal triangle impressed, with smooth surface and 2–4 short longitudinal striae, shiny. Frontal carinae short, slightly extending beyond frontal lobes. Frons narrow, in the narrowest part ~ 0.28× as wide as head width. Antennal fossa deep, not surrounded by semicircular striae, surface smooth and shiny. Head mostly without background microreticulation or with strongly diffused microreticulation, micropunctate with very small and sparse pits, strongly shiny. Frons mostly or completely lacking longitudinal striae, sometimes few striae laterally, with or without very narrow short shallow median sulcus, gena with short longitudinal striae, area behind rather smooth or with indistinct microreticulation, without striation (Fig. 10). The whole head surface covered with short and sparse, but clearly visible white appressed pubescence. Frontal lobes with single long erect seta, and frons behind frontal carinae with single long erect seta and laterally to ocellar cavity with two regular rows of three or four setae, often broken in mature specimens. Vertex with 12–20 long erect setae on almost whole occipital region including occipital corners, often with several short decumbent setae between the large setae, sides of the head and gena without standing setae, occasionally gena with single short semierect seta. Ventral part of the head and inner margin of mandibles with numerous, long erect setae, partly forming a J-shaped psammophore. Antennal scape short, in frontal view almost straight, only apically slightly curved, without preapical constriction, 0.68–0.74× as long as the width of the head; base of scapus moderately extended, outer angle acute, inner angle angulate. Funiculus distinctly longer than scape, pedicel moderately elongated, ~ 2.2× as long as wide at apex, not flattened dorsoventrally, ~ 0.74× as long as segments 2 and 3 combined and 1.5× as long as segment 2 (Fig. 10). Surface of the scape with diffused microreticulation, shiny, covered with long and sparse, white, decumbent to subdecumbent hairs. Mandibles rounded, with deep striae, surface shiny with a few long and short yellow setae, cutting edge in large majors without teeth or with serrulate edge. ***Mesosoma*.** Moderately long, 2.0–2.3× as long as wide. Promesonotum regularly convex in profile with pronotum not bulging above mesonotal plate (Figs 2, 4–8), pronotal sides regularly rounded (Fig. 10). Propodeum positioned lower than promesonotum, flat to very slightly convex anteriorly then subangulate (Figs 2, 9) to rounded posteriorly (Fig. 8), posterior slope oblique, flat, distinctly concave thus propodeal angle distinct, forming angulate tubercle (Fig. 30). Pronotum anteriorly and dorsally with transverse rugae and diffusely microreticulate interspaces but shiny, often anterolateral corners of pronotum with diffused rugae, sides of pronotum in pale forms with fine semicircular striae, often partly diffused, in dark forms with more evident striae, interspaces indistinctly microreticulate but shiny. Elevated dorsal plate of mesonotum mostly without rugae, sometimes anteriorly with short longitudinal rugae and laterally with slightly irregular surface, mesopleura with sharp perpendicular rugae and strong microreticulation. Propodeum dorsally, on dorsal half of sides and on posterior face with sharp transverse rugae, in metapleural area with sharp longitudinal rugae, interspaces with diffused microreticulation, on metapleuron and posterior face of propodeum partly smooth and shiny. Vestiture and setation of mesosoma sparse, pronotum with 8–12 long, yellow erect setae, the longest with length 0.285, sides of the pronotum without suberect setae, mesonotum anteriorly with 8–10 and posteriorly 5–8 long erect setae, metapleuron in posterolateral corners with short subdecumbent setae, propodeum dorsally with one pair of long erect setae and often 1–3 setae laterally, often broken in mature specimens. ***Petiole*.** Elongate, with long pedicel and moderately high triangular node, thin, PI 1.3–1.4, pedicel, and base of node with distinct reticulate sculpture, anterior face concave with diffused microreticulation, shiny, sides of node strongly microreticulate with few rugosities. Top of petiole angulate; upper margin and sides with 6–8 erect setae. ***Postpetiole*.** Rounded in profile, globular in dorsal view, 1.3–1.4× as wide as the petiole, surface microreticulate, and often with short longitudinal striae, with 6–8 setae on top and 10–12 long erect setae along base. ***Gaster*.** Whole surface of first tergite with diffused but clearly visible background microreticulation, surface shiny, but sometimes the microreticulation more distinct especially in the largest majors or the darkest specimens, covered with extremely sparse and short appressed hairs and at base usually with several short erect setae, sometimes posterior half of first gastral tergite with few erect setae, in north-eastern populations (Bulgaria, Greek Thraki) base of gaster is less setose than in populations from Epirus and Ionian Islands, only occasionally whole first tergite without setae; second tergite with several long erect setae. ***Legs*.** Moderately elongate, femora distinctly swollen centrally, tibiae moderately widened apically, mid and hind tarsi longer than tibiae. Whole surface of femora diffusely microreticulated, dorsally and laterally covered with moderately sparse and long, decumbent and subdecumbent and ventrally semierect to erect setae. Surface of tibiae covered with sparse and long decumbent to semierect setae.

Minor workers (*n* = 5): HL: 0.997–1.135 (mean 1.067); HW: 0.921–1.063 (mean 1.014); SL: 0.849–0.952 (mean 0.894); EL: 0.198–0.238 (mean 0.217); WL: 1.365–1.571 (mean 1.476); MW: 0.611–0.698 (mean 0.668); PSL: 0.146–0.162 (mean 0.153); PW: 0.211–0.254 (mean 0.238); PPW: 0.309–0.349 (mean 0.329); HL/HW: 1.026–1.082 (mean 1.054); SL/HW: 0.836–0.922 (mean 0.883); WL/MW: 2.164–2.251 (mean 2.210); EL/HL: 0.182–0.215 (mean 0.204); PSL/HW: 0.143–0.158 (mean 0.151); PPW/PW: 1.299–1.464 (mean 1.383).

***Colour*.** As coloured as major workers, but pale specimens are more frequent than in majors (Figs 3, 4, 6, 7). ***Head*.** Slightly more elongated and more rounded in frontal view than in major workers, 1.03–1.08× as long as wide, softly converging anterad and posterad, behind eyes more regularly rounded, occipital margin of the head slightly convex (Fig. 11). Clypeus as sculptured as in majors, shiny. Frons mostly smooth and shiny, without or with very short striae laterally, gena with shorter rugae than in majors. Background microreticulation of head usually completely reduced thus surface appear strongly smooth and shiny. ***Mesosoma*.** Slightly slimmer than in majors, WL/MW ratio ~ 2.2. Pronotal surface mostly without rugae, only with diffused microreticulation. Sculpture of mesonotum and propodeum as in majors. Propodeal angle less marked than in majors (Fig. 6). Setation and vestiture of mesosoma as in majors but with lower number of setae, propodeum often without standing setae). ***Petiole and postpetiole*.** As in major workers but surface with partly reduced reticulation and striae and with smaller numbers of erect setae. ***Gaster*.** Shinier than in majors, with background microreticulation often completely diffused, especially in posterior half of first gastral tergite. Rest of characters as in major workers.

Gynes (*n* = 2): HL: 1.900–1.943 (mean 1.922); HW: 1.990–2.120 (mean 2.055); SL: 1.508–1,614 (mean 1.561); EL: 0.508–0.510 (mean 0.509); WL: 3.750–3.796 (mean 3.773); MW: 1.830–1.957 (mean 1.894); PSL: 0.405–0.410 (mean 0,408); PW: 0.603–0.642 (mean 0.623); PPW: 0.778–0.835 (mean 0.807); HL/HW: 0.917–0.955 (mean 0.936); SL/HW: 0.758–0.761 (mean 0.760); WL/MW: 1.940–2.049 (mean 1.994); EL/HL: 0.262–0.267 (mean 0.265); PSL/HW: 0.193–0.204 (mean 0.198); PPW/PW: 1.290–1.301 (mean 1.295).

**Figures 12, 13.**
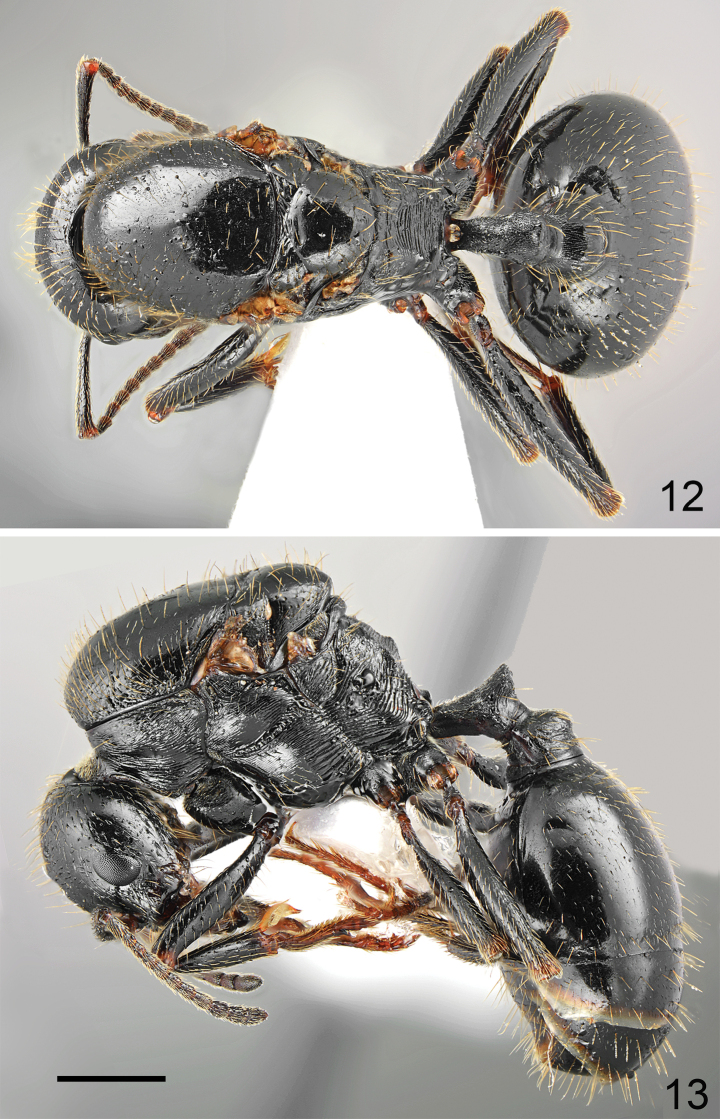
Gyne of *Messoratanassovii* Atanassov, 1982, population from Epirus **12** dorsal **13** lateral. Scale bar: 1 mm.

**Figure 14.**
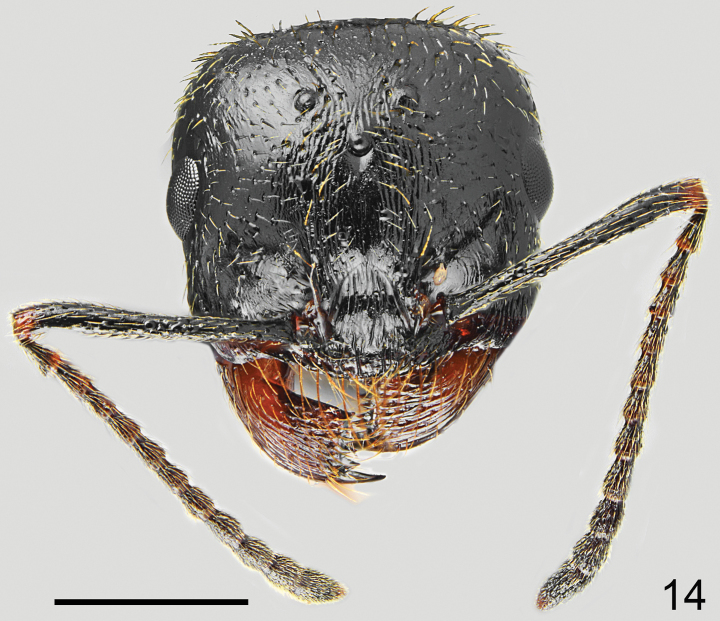
Head of gyne of *Messoratanassovii* Atanassov, 1982, population from Epirus. Scale bar: 1 mm.

***Colour*.** Whole body black, including head, petiole and postpetiole, and gaster (gyne from Lefkada) or almost entirely black, except for anterior margin of genae, mandibles, and antennas, which are reddish brown (gyne from Bulgaria). Legs predominantly black except reddish knee and part reddish yellow tarsi (gyne from Lefkada) (Figs 12, 13) or with black coxa and femur, reddish brown knees, tibiae, and tarsi (gyne from Bulgaria). ***Head*.** Subrectangular, ~ 0.94× as long as wide, sides below eyes subparallel, above eyes subparallel then rounded, posterior margin shallowly concave (Fig. 14). Anterior clypeal margin convex, without median emargination, with a row of 12 long marginal, six submarginal and four medial yellowish setae, the longest as long as length of clypeus. Clypeus without appressed pubescence. Surface of the clypeus irregular, centrally with longitudinal rugae and with median keel, interspaces microreticulate but shiny (Fig. 14). Eyes moderately large, EL/HL ratio ~ 0.27 and broadly oval and approximately as long as the gena. Frontal triangle impressed, with smooth surface and several longitudinal striae, shiny. Frontal carinae short, slightly extending beyond frontal lobes. Frons narrow, in the narrowest part 0.25–0.32× as wide as head width. Antennal fossa deep, surrounded by diffused semicircular striae, surface smooth or with diffused microreticulation, shiny. Head mostly smooth and shiny with sparse pits. Frons on almost whole surface with well-marked longitudinal striae and with very narrow, short, shallow median sulcus, gena with short longitudinal striae, area behind eyes with diffused microreticulation, without striation. Surface covered with short and sparse white appressed pubescence. Frontal lobes with single long erect seta, and frons behind frontal carinae up to posterior ocelli with two irregular rows of several long erect setae. Vertex with numerous long erect setae placed in whole occipital area but with the most numerous setae in occipital corners. Ventral part of the head with several, long, partly J-shaped erect setae. Antennal scape short, in frontal view almost straight only apically slightly curved, without preapical constriction, 0.76× as long as the width of the head; base of scapus moderately extended, outer and inner angle angulate. Funiculus distinctly longer than scape, pedicel moderately elongated, 2.2–2.5× as long as wide at apex, not flattened dorsoventrally, 0.69–0.82× as long as segments 2 and 3 combined and 1.2–1.46× as long as segment 2 (Fig. 14). Surface of the scape with diffused microreticulation, shiny, covered with long and sparse, white, decumbent to suberect hairs. Mandibles rounded, with deep striae, surface shiny with a few long and short yellow setae, cutting edge with large, sharp teeth (Fig. 14). ***Mesosoma*.** Stout, approximately twice as long as wide. Pronotum very short and not visible from above. Scutum mostly flat and only anteriorly slightly bulging (Fig. 13). Surface of scutum smooth and shiny, without striation or rugae. Scutellum slightly convex, 1.2× as wide as long, surface smooth and shiny (Fig. 12). Propodeum on sides with sharp carina forming at propodeal angle sharp elevation but without denticle (Fig. 13). Pronotum anteriorly and laterally with transverse rugae and diffusely microreticulate interspaces but shiny, anepisternum and katepisternum with fine transverse rugae, finer anteriorly and sharper posteriorly, in the middle of katepisternum rugae partly diffused, interspaces mostly smooth and shiny. Propodeum dorsally, on dorsal half of sides and on posterior face with sharp transverse rugae, in metapleural area with sharp longitudinal rugae, interspaces with distinct microreticulation. Vestiture and setation of scutum sparse, only in anterior half with very sparse decumbent hairs, rest of surface with erect setae, denser in anterolateral corners and gradually sparser posterad, the longest setae with length 0.3, scutellum with only two setae centrally and several setae laterally, anepisternum with only few subdecumbent setae, katepisternum with less than 20 subdecumbent to semierect setae. ***Petiole*.** Elongate, with long pedicel and moderately high triangular node, thin, PI 1.5, pedicel, and base of node with distinct reticulate sculpture, anterior face of node with diffused microreticulation, shiny, sides of node microreticulate and posterior face of node microreticulate, without rugae or striation. Top of petiole sharply angulate, with six erect setae. ***Postpetiole***. Rounded in profile, globular in dorsal view, 1.3× as wide as the petiole, whole surface with 26 long erect setae. ***Gaster*.** Whole surface of first tergite with fine but clearly visible background microreticulation, surface shiny, covered with sparse and short semierect and erect setae, the longest with length 0.190; second tergite with two rows of erect setae, 1/3 longer than setae on first tergite. ***Legs*.** Moderately elongate, femora distinctly swollen centrally, tibiae moderately widened apically, mid and hind tarsi shorter than tibiae. Whole surface of femora diffusely microreticulated, dorsally and laterally covered with moderately sparse and long, decumbent and subdecumbent and ventrally semierect to erect setae. Surface of tibiae covered with sparse and long decumbent to semierect setae, fore femora ventrally with row of 12 long and moderately long setae.

##### Comparative remarks.

*Messoratanassovii* with mean HW of the largest workers 1.98 (max 2.30) and HL 1.90 (max 2.13) belongs to the small members of the *M.instabilis* group in the Balkans and is distinctly smaller than members of the *Messorwasmanni* complex with mean HW of the largest workers > 2.65 (max 2.95) and HL > 2.46 (max 2.68). It is slightly larger than both new species from Cyclades: *M.danaes* sp. nov. and *M.veneris* sp. nov. that have mean HW 1.78 (max 1.91) and 1.89 (max 2.04) respectively. *M.danaes* sp. nov. differs also in body always uniformly black while in *M.atanassovii* most specimens have mesosoma mostly or partly red and specimens with body predominantly black represent less than 5% of population. Both *M.danaes* sp. nov., and *M.veneris* sp. nov. have small numbers of large setae in occipital part of head, usually 1–6 (up to 8), while *M.atanassovii* has 12–20 setae on occiput. *Messorkardamenae* sp. nov. has the same range of colour variation of mesosoma as *M.atanassovii* (from predominantly red to mostly brown or partly black) but it differs in larger body size with mean ML of the largest workers 2.92 (max 3.25), HW 2.63 (max 2.85) and HL 2.41 (max 2.55) while in *M.atanassovii* mean ML is 2.40 (max 2.73), HW 1.98 (max 2.30) and HL 1.90 (max 2.13) respectively. *Messoratanassovii* has relatively longer antennal scapes in major workers with SL/HW 0.68–0.74 (mean 0.721) while in *M.kardamenae* sp. nov. SL/HW is 0.64–0.68 (mean 0.659) respectively. In *M.atanassovii*, the first gastral tergite usually has very short and sparse erect setae (mostly limited to basal third of the first tergite) but sometimes erect setae are present also in posterior half of its surface while *M.kardamenae* sp. nov. has first gastral tergite always lacking erect setae. *Messorcreticus* appears to be the most similar to *M.atanassovii* but it differs in less numerous erect setae on posterior head, stronger sculpture on propodeum that is entirely covered with thick and sparser rugae, and its dorsum does not bear reduced sculpture or smooth patches. Both species are separated geographically, *M.atanassovii* is northern species noted from Bulgaria and north and western Greek provinces (Epirus, Ionian Islands, Eastern Macedonia, and Thraki and Central Macedonia) while *M.creticus* is southern species and occurs only in Crete. From other species of *M.semirufus* complex known from the eastern part of the Mediterranean basin, only similarly sized and coloured specimens of *M.intermedius* appear similar but they differ in occipital part of head without or at most with two erect setae, and the lack of erect setae on the first gastral tergite.

##### Biological notes.

Recorded from lowland and highland open habitats from sea level to an altitude 1010 m. In Greece, it was noted in mountain pastures, sands near marshes close to the seacoast, on a beach, and in a ruderal area in a tourist resort. At the same time, in Bulgaria, it was frequently collected along dirt roads, sometimes water channels and riverbeds in the area. Nests were observed in sandy areas directly in the ground. Workers built around the nest’s entrance a low mound of sand without plant remains or herb seeds. Contrary to *Messorwasmanni* Krausse occurring in the same areas, workers did not tread paths in the ground and penetrated the vicinity of the nest at a distance of only 2 meters from the entrance to the nest. At temperatures less than 28 °C, workers were also active in the middle of the day.

#### ﻿ Messor danaes

Taxon classificationAnimaliaHymenopteraFormicidae

Salata, Georgiadis & Borowiec sp. nov.

4F188A12-3D61-50C5-8746-6EEC90F48F7F

https://zoobank.org/5BCEA092-C0C8-4BD4-89D4-44848EA29A40

**Figures 15, 16.**
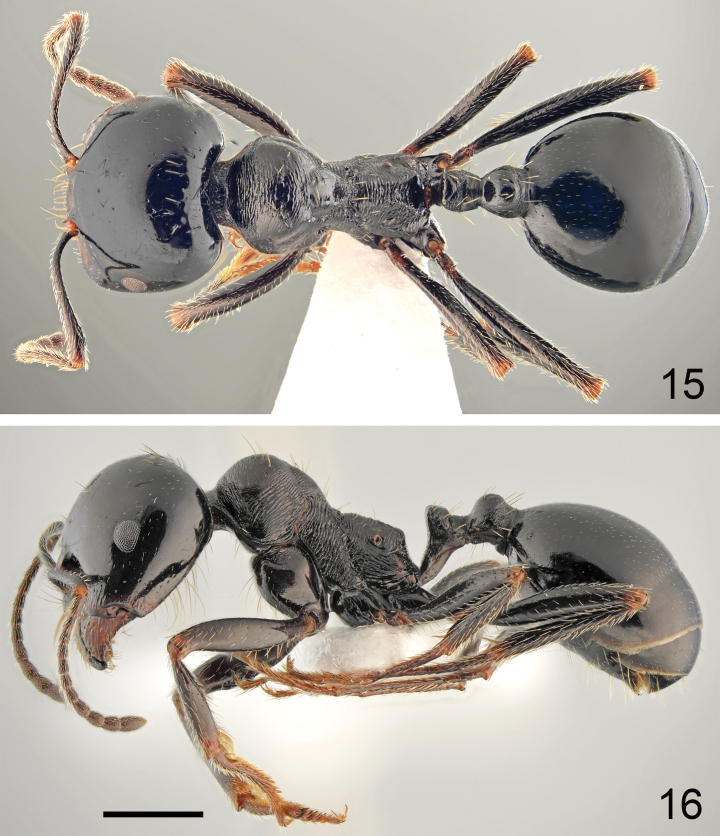
Major worker of *Messordanaes* Salata, Georgiadis & Borowiec, sp. nov., holotype **15** dorsal **16** lateral. Scale bar: 1 mm.

**Figures 17, 18.**
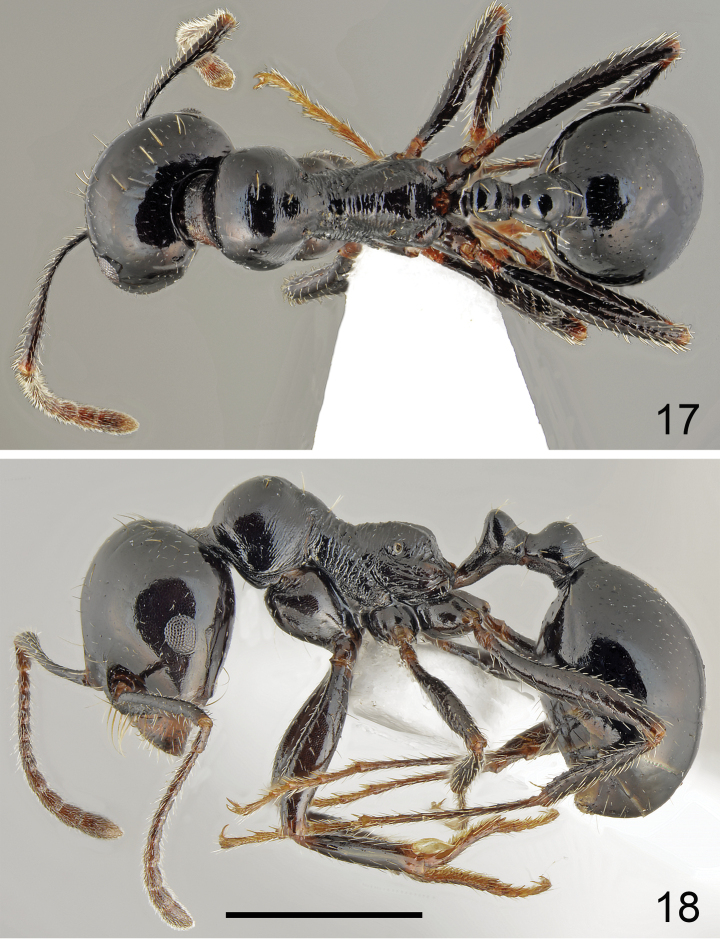
Minor worker of *Messordanaes* Salata, Georgiadis & Borowiec, sp. nov. **17** dorsal **18** lateral. Scale bar: 1 mm.

**Figures 19, 20.**
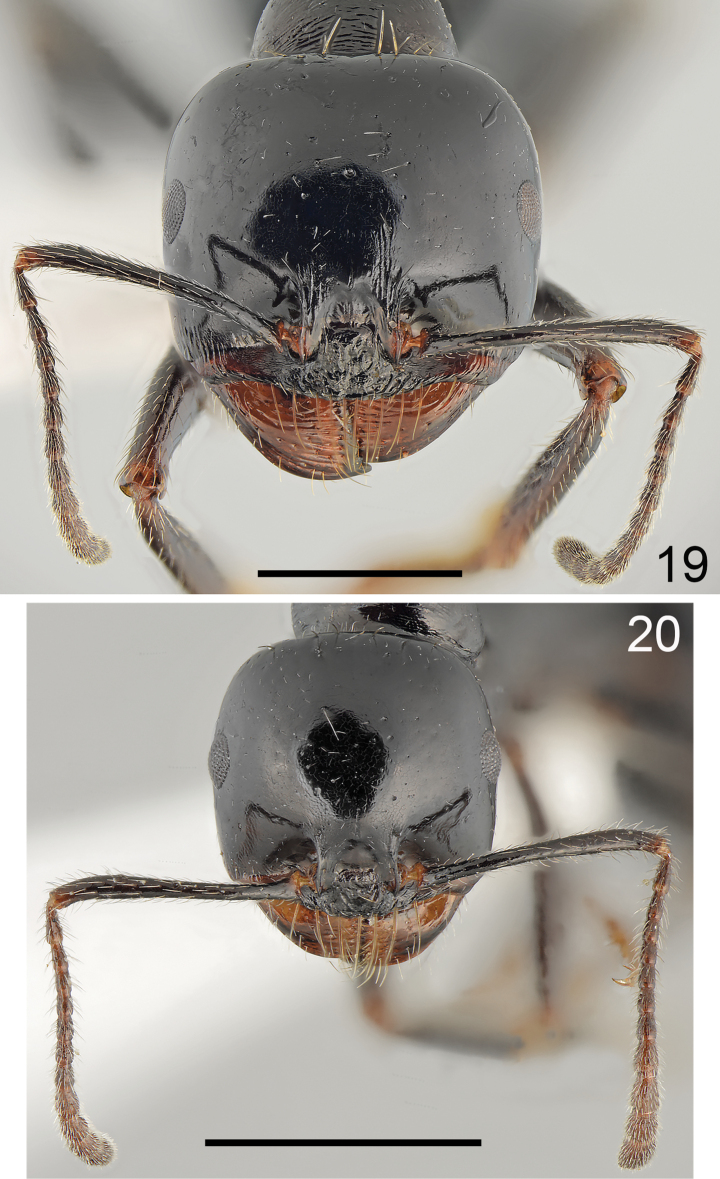
Head of *Messordanaes* Salata, Georgiadis & Borowiec, sp. nov. **19** major worker **20** minor worker. Scale bar: 1 mm.

Figs 15–16 , 17, 18 , 19–20

##### Type material.

***Holotype*** major worker (pin): Greece, Cyclades, Serifos, | Gyftica Helicopter Airport | loc. 1, 206 m | 37.1630, 24.4839, 08.06.2022 S.| Salata CYC131 || Collection MNHW | Formicidae | MNHW–GR03221 (MNHW); ***Paratypes***: 7 major, 10 medium, 5 minor workers (pin), the same data as for holotype (MNHW).

##### Other material examined.

15w. (EtOH): the same data as holotype (MNHW).

##### Description.

***Measurements*.** Major workers (*n* = 5): HL: 1.524–1.880 (mean 1.729); HW: 1.548–1.910 (mean 1.980); SL: 1.214–1.368 (mean 1.321); EL: 0.270–0.333 (mean 0.309); WL: 1.980–2.367 (mean 2.181); MW: 0.963–1.139 (mean 1.066); PSL: 0.230–0.270 (mean 0.250); PW: 0.333–0.413 (mean 0.375); PPW: 0.412–0.524 (mean 0.475); HL/HW: 0.957–0.984 (mean 0.973); SL/HW: 0.716–0.784 (mean 0.745); WL/MW: 2.003–2.078 (mean 2.045); EL/HL: 0.175–0.186 (mean 0.179); PSL/HW: 0.130–0.154 (mean 0.141); PPW/PW: 1.237–1.307 (mean 1.266).

***Colour*.** Whole body black, only in few of the largest major workers anterolateral corners of pronotum with dark red discoloration and frontal triangle reddish (Figs 15, 16). Legs dark brown to black, only apex of trochanters, knee and ventral side of tarsi yellowish to yellowish brown, occasionally coxa partly reddish brown. Mandibles reddish to reddish brown. Antennal scapes black with yellowish brown apex, funicle usually black basally then gradually paler apically with last segments brown, sometimes whole funicle brown (Fig. 19). ***Head*.** Subrectangular, 0.96–0.98× as long as wide, sides below eyes slightly converging anterad, above eyes subparallel then softly convex, posterior margin shallowly concave (Fig. 19). Anterior clypeal margin straight, without median emargination, with a row of ten long marginal yellowish setae, the longest as long as the length of clypeus. Clypeus without appressed pubescence, laterally with three or four erect setae and long erect seta close to lateral margin of central plate. Surface of the clypeus irregular, with short rugae, median clypeal carinae absent, interspaces smooth and shiny (Fig. 19). Eyes small and broadly oval, 0.5–0.6× as long as the gena. Frontal triangle shiny, impressed, with microreticulate surface and 2–4 short longitudinal striae. Frontal carinae short, slightly extending beyond frontal lobes. Frons narrow, in the narrowest part ~ 0.26× as wide as head width. Antennal fossa deep, not surrounded by semicircular striae, surface smooth or with diffused microreticulation, shiny. Head mostly smooth and shiny, only with diffused background microreticulation and very sparse minute pits, frons along frontal carinae with short striae and with very narrow and shallow median sulcus, usually with large pit behind the median sulcus, gena only close to anterior margin with short longitudinal striae, area behind eyes as smooth as rest of head, without striation. Surface covered with extremely short and sparse, hardly visible white appressed pubescence. Frontal lobes with single long erect seta, and frons behind frontal carinae with one or two long erect setae and centrally with two rows of two or three setae often broken in mature specimens. Occipital area with 6–8 (at most 10) long erect setae placed closer to median impression than in occipital corners, which are without or with single semierect setae, sides of the head, and gena without standing setae, occasionally gena with a single short standing seta. Ventral part of the head with numerous, long erect setae, partly forming a J-shaped psammophore. Antennal scape short, in frontal view almost straight only apically slightly curved, without preapical constriction, 0.72–0.78× as long as the width of the head; base of scapus moderately extended, outer angle acute, inner angle forms small obtuse lobe. Funiculus distinctly longer than scape, pedicel moderately elongated, ~ 2.1× as long as wide at apex, not flattened dorsoventrally, ~ 0.75× as long as segments 2 and 3 combined and 1.3× as long as segment 2 (Fig. 19). Surface of the scape with diffused microreticulation, shiny, covered with long and sparse white subdecumbent to suberect hairs. Mandibles rounded, with deep striae, surface shiny with a few long and short yellow setae, cutting edge in large majors without teeth or with serrulate edge. ***Mesosoma*.** Moderately long, 2.0–2.1× as long as wide. Promesonotum not regularly convex in profile with pronotum slightly bulging above mesonotal plate (Fig. 16), pronotal sides regularly rounded (Fig. 15). Propodeum positioned lower than promesonotum, flat anteriorly then angulate posteriorly, angulation margined by short, thick carina lateral but never forms distinct spine (Fig. 16). Pronotum anteriorly and dorsally with transverse rugae and diffusely microreticulate interspaces but shiny, sides with distinct oblique striae, and more distinctly microreticulate interspaces but shiny. Elevated dorsal plate of mesonotum mostly diffusely microreticulate, smooth and shiny, posterior part of mesonotal dorsum irregular, sides and mesopleura with sharp perpendicular rugae and strong microreticulation. Propodeum dorsally, on dorsal half of sides and on posterior face with sharp transverse rugae, in metapleural area with sharp longitudinal rugae, interspaces with diffused microreticulation only on metapleuron smooth and shiny. Vestiture and setation of mesosoma sparse, pronotum with 4–8 long, yellow erect setae, the longest with length 0.238, sides of the pronotum with a few short suberect setae, mesonotum anteriorly with four and posteriorly 6–8 long erect setae, metapleuron in posterolateral corners with short subdecumbent setae, propodeum with one or two pairs of long erect setae often broken in mature specimens. ***Petiole*.** Elongate, with long pedicel and moderately high triangular node, thin, PI 1.6–1.7, pedicel, and base of node with distinct reticulate sculpture, anterior face of node smooth and shiny, sides of node microreticulate and posterior face of node microreticulate and before the top with two or three transverse rugae. Top of petiole angular, upper margin and sides with 10–12 erect setae. ***Postpetiole*.** Rounded in profile, globular in dorsal view, 1.2–1.3× as wide as the petiole, whole surface with 14–16 long erect setae and few suberect hairs on top. ***Gaster*.** Whole surface of first tergite smooth and shiny but with marked, partly diffused background microreticulation, covered with extremely sparse and short appressed hairs, completely without erect setae; second tergite without long erect setae but usually with two short and often two additional and very short semierect setae. ***Legs*.** Moderately elongate, femora distinctly swollen centrally, tibiae moderately widened apically, mid and hind tarsi longer than tibiae. Whole surface of femora diffusely microreticulated, dorsally and laterally covered with moderately sparse and long, decumbent and subdecumbent, and ventrally semierect to erect setae. Surface of tibiae covered with sparse and long decumbent to semierect setae.

Minor workers (*n* = 5): HL: 1.003–1.254 (mean 1.104); HW: 0.981–1.238 (mean 1.074); SL: 0.913–1.060 (mean 0.965); EL: 0.222–0.251 (mean 0.229); WL: 1.405–1.730 (mean 1.530); MW: 0.667–0.828 (mean 0.728); PSL: 0.175–0.206 (mean 0.189); PW: 0.246–0.287 (mean 0.265); PPW: 0.324–0.393 (mean 0.350); HL/HW: 1.013–1.047 (mean 1.028); SL/HW: 0.856–0.931 (mean 0.901); WL/MW: 2.016–2.183 (mean 2.102); EL/HL: 0.197–0.215 (mean 0.207); PSL/HW: 0.164–0.181 (mean 0.174); PPW/PW: 1.288–1.369 (mean 1.318).

***Colour*.** As coloured as major workers, without red discoloration on pronotum (Figs 17, 18). ***Head*.** Slightly more elongated and more rounded in frontal view than in major workers, 1.01–1.05× as long as wide, softly converging anterad and posterad, behind eyes more regularly rounded, occipital margin of the head slightly convex (Fig. 20). Clypeus as sculptured as in majors, shiny. Frons mostly smooth and shiny, without or with remnants of striae, gena without rugae only anterior margin with short striae. ***Mesosoma*.** Slightly slimmer than in majors, WL/MW ratio ~ 2.2. Pronotal surface mostly smooth and shiny or only with remnants of rugae. Sculpture of mesonotum and propodeum as in majors. Setation and vestiture of mesosoma as in majors but with lower number of setae, often pronotum without or only with two setae and mesonotum with only four erect setae (Fig. 18). ***Petiole and postpetiole*.** As in major workers but surface with mostly reduced reticulation and rugae and with smaller numbers of erect setae. ***Gaster*.** Smoother and shinier than in majors, with microreticulation mostly diffused. Rest of characters as in major workers.

##### Comparative remarks.

*Messordanaes* sp. nov. with *M.veneris* sp. nov. are the smallest Balkan members of the *M.semirufus* complex. The largest majors have HW and HL < 2.0 mm (only two of the studied specimens have HW > 2.0 mm but < 2.1 mm). They are clearly characterised by low numbers of occipital setae, always fewer than nine. *Messorveneris* sp. nov. clearly differs from *M.danaes* sp. nov. in bicoloured body with completely or predominantly red mesosoma while in *M.danaes* sp. nov. mesosoma is entirely black. *Messorveneris* sp. nov. has very distinct and regular background microreticulation of head and first gastral tergite while in *M.danaes* sp. nov. head and gaster are smooth and shiny, only with diffused and, especially on head, hardly visible background microreticulation. Rare dark (predominantly dark brown to almost black) forms of *M.atanassovii*, *M.creticus* and *M.kardamenae* sp. nov. clearly differ from *M.danaes* sp. nov. in numerous occipital setae (7–20) spread on almost the whole occipital region while *M.danaes* sp. nov. has the smallest number of occipital setae in the entire complex (6–8, at most 10, grouping more centrally on vertex than in occipital corners). From other species of the *M.semirufus* complex known from the eastern part of the Mediterranean basin, only *M.ebeninus* has uniformly black body but it differs in larger body size with HW in the largest majors up to 2.6 mm and in more evident head sculpture with longitudinal striation present in the whole frontal area and around the antennal fossae. From other species of the *M.semirufus* complex known from the western part of the Mediterranean basin, only *M.bouvieri* Bondroit, 1918 is similar due to the almost uniformly black body but differs in more evident head sculpture with longitudinal striation present in whole frontal area, around antennal fossae and above rugae on gena. The dark populations of Italian *M.minor* spp. *calabricus* Santschi, 1927 differs in presence of several very short and sparse erect setae on the first gastral tergite, and the number of occipital setae on the head distinctly exceeding ten.

##### Biology.

The nest sample was collected in phrygana located in the central, mountainous part of the island. The nest was located under a stone, close to a dirt road.

##### Etymology.

Named after Danae (Greek: Δανάη), an Argos princess, and mother of the hero Perseus. Based on the mythology, the oracle of Delphi announced to King Argos that his daughter’s son would kill him. Thus, the king, to escape his destiny, decided to cast Danae and Perseus into the sea in a wooden chest hoping for their inevitable death. However, both survived and were washed ashore on the island of Serifos, the type locality for *Messordanaes*. The epithet is genitive.

#### ﻿ Messor kardamenae

Taxon classificationAnimaliaHymenopteraFormicidae

Salata & Borowiec sp. nov.

FCB6665B-CE71-5406-9604-C5FF62E566D5

https://zoobank.org/50D8DDBD-B234-4B2E-8134-9EB62E373CE0

Figs 21–22 , 23, 24 , 25, 26 , 27–28

Messor cf. semirufus : Borowiec et al. 2021: 21.

##### Type material.

***Holotype*** major worker (pin): GREECE, Dodecanese, Kos | Kardamena city, 7m | 36.78363 N / 27.14107 E | 9 VII 2015, S. Salata || Collection L. Borowiec | Formicidae | LBC-GR01961 (MNHW). ***Paratypes***: 6 major, 6 medium, 1 minor workers (pin), the same data as for holotype (MNHW).

##### Other material examined.

Greece • 32w. (EtOH): the same data as holotype (MNHW). • 1w. (pin): Dodecanese, Nisyros, Loutra; 36.61127, 27.15559; 27 m; 29 Sep 2019; leg. C. Lebas (MNHW). • 2w. (pin); Dodecanese, Nisyros, Castro; 36.60437, 27.13181; 99 m; 29 Sep 2019; leg. C. Lebas (MNHW). • 2w. (pin); Dodecanese, Nisyros, Stefanos Volcano 2; 36.57561, 27.15592; 226 m; 30 Sep 2019; leg. C. Lebas (MNHW). • 7w. (4w. pin, 3w. EtOH): Dodecanese, Nisyros, Stefanos Volcano 3; 36.57926, 27.16698; 113 m; 30 Sep 2019; leg. C. Lebas (MNHW). • 1w. (EtOH); Dodecanese, Nisyros, Pali; 36.61924, 27.16816; 35 m; 29 Sep 2019; leg. C. Lebas (MNHW). • 8w. (pin); Dodecanese, Rhodes, Kiotari at night; 36.02, 27.57; 12 m; 09 Jul 2008; leg. L. Borowiec (MNHW). • 2w. (pin); Dodecanese, Rhodes, Gennadi-Chochlakas rd.; 35.95, 27.8666; 36 m; 12 Jul 2008; leg. L. Borowiec (MNHW). • 10w. (pin); Dodecanese, Rhodes, Pefkos env.; 36.06, 28.05, 50 m; 18 Sep 2011; leg. G. Ruzzante (MNHW). • 2w. (EtOH); Dodecanese, Tilos, Livadia Beach; 36.4123, 27.38891; 3 m; 26 Sep 2019; leg. C. Lebas (MNHW). • 2w. (EtOH); Dodecanese, Tilos, Mikro Chorio; 36.42243, 27.37098; 246 m; 24 Sep 2019; leg. C. Lebas (MNHW).

##### Description.

***Measurements*.** Major workers (*n* = 5): HL: 2.333–2.550 (mean 2.409); HW: 2.500–2.850 (mean 2.633); SL: 1.660–1.833 (mean 1.735); EL: 0.444–0.484 (mean 0.459); WL: 2.700–3.250 (mean 2.923); MW: 1.365–1.540 (mean 1.427); PSL: 0.317–0.428 (mean 0.354); PW: 0.432–0.619 (mean 0.495); PPW: 0.611–0.833 (mean 0.687); HL/HW: 0.895–0.933 (mean 0.916); SL/HW: 0.643–0.676 (mean 0.659); WL/MW: 1.978–2.110 (mean 2.048); EL/HL: 0.188–0.194 (mean 0.190); PSL/HW: 0.124–0.150 (mean 0.134); PPW/PW: 1.343–1.435 (mean 1.390).

**Figures 21, 22.**
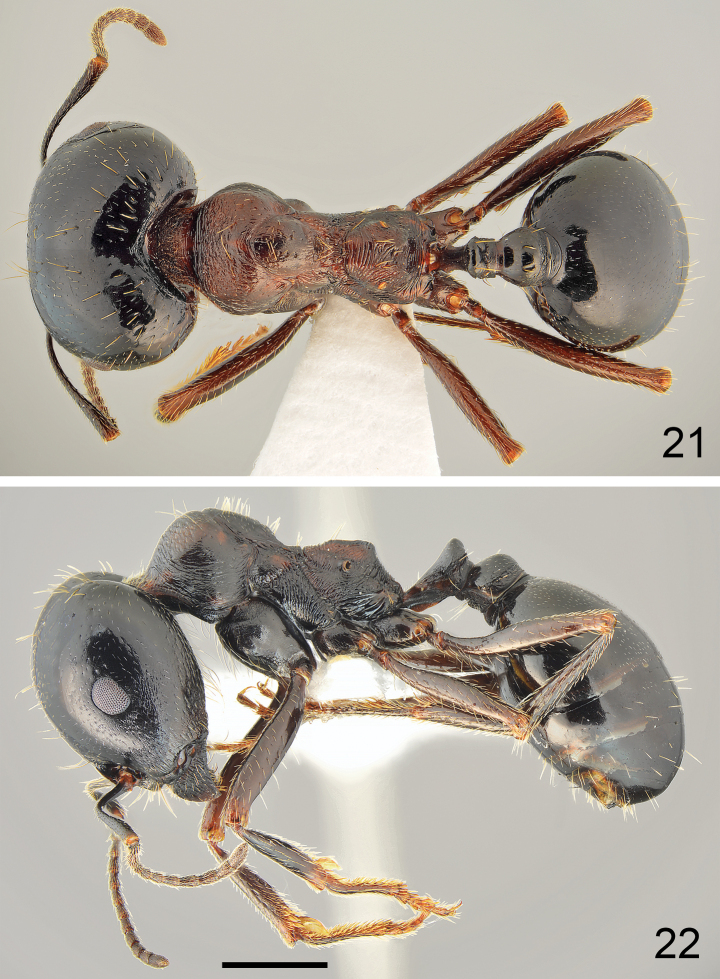
Major worker of *Messorkardamenae* Salata & Borowiec, sp. nov., holotype from Kos **21** dorsal **22** lateral. Scale bar: 1 mm.

***Colour*.** Variable, head usually completely black, only mandibles reddish to reddish brown and frontal triangle reddish, often anterior margin of gena also reddish, occasionally in medium size majors workers vertex with reddish discoloration. Mesosoma from predominantly red to predominantly brown to black with reddish patches. In the darkest populations sides of mesosoma mostly brownish black with reddish discolorations on sides of pronotum and upper parts of mesonotum and propodeum but with reddish to reddish-brown dorsum (Figs 21 [holotype], 22 [paratype from the same nest]). In pale populations mesosoma completely reddish with indistinct obscure discolorations on mesonotum (Fig. 25, population from Rhodes). Petiole and postpetiole reddish, reddish brown, brown, or black. Gaster in mature specimens black, only tergites with reddish or yellowish transparent hind margins, in premature specimens at base sometimes with reddish discoloration. Legs in dark specimens predominantly brown, sometimes coxa black with yellowish apices, trochanters brown with yellowish apical margin, femora brown with yellowish knee, tibiae brown, tarsi brown dorsally and yellowish ventrally (Figs 21, 22). In pale specimens coxa, trochanters, femora and tibiae reddish brown with yellow apices and tarsi yellow (Figs 25, 26). Antennal scapes mostly dark brown to black with yellowish brown apex, funicle usually completely brown or dark brown basally and yellowish brown apically (Figs 27, 28). ***Head*.** Subrectangular, 0.90–0.93× as long as wide, sides below eyes softly converging anterad, above eyes subparallel then softly rounded, posterior margin deeply concave (Fig. 22). Anterior clypeal margin straight, without median emargination, with a row of ten long marginal, yellowish setae, the longest as long as length of clypeus. Clypeus without appressed pubescence, laterally with two pairs of long and one or two short erect seta and long erect seta close to the middle of lateral margin of central plate. Surface of the clypeus on sides irregular, in centre mostly regular, only on sides with short longitudinal one or two striae, clypeal alae with few irregular rugae, no median keel, interspaces diffusely microreticulate, shiny (Fig. 27). Eyes small and broadly oval, 0.6–0.7× as long as the gena. Frontal triangle impressed, with 4 striae converging anteriorly, surface smooth and shiny. Frontal carinae short, slightly extending beyond frontal lobes. Frons narrow, in the narrowest part ~ 0.26× as wide as head width. Antennal fossa deep, surrounded by fine, sometimes partly diffused, semicircular striae, surface smooth or with diffused microreticulation, shiny. Head mostly with diffused and invisible background microreticulation but with numerous pits, small and sparse on sides of head, moderately coarse and partly elongate above frontal striation, surface shiny. Frons in the middle with clearly marked longitudinal striae and with very narrow, short, and shallow median sulcus, but without ocellar cavity behind the median sulcus, gena with short longitudinal striae, area behind eyes only with pits, without striation. Surface covered with short and sparse, clearly visible white appressed pubescence. Frontal lobes with single long erect seta, and frons behind frontal striae with a pair of long erect setae and on vertex with two irregular rows of three or four long setae and often with additional 2–4 short setae, often broken in mature specimens. Occipital area with 7–12 erect setae 0.5× as long, grouped mostly in occipital corners, sides of head and gena without standing setae. Ventral part of the head and inner margin of mandibles with numerous, long erect setae, partly forming a J-shaped psammophore. Antennal scape short, in frontal view almost straight only apically slightly curved, without preapical constriction, 0.64–0.68× as long as the width of the head; base of scapus moderately extended, both outer and inner angle angulate. Funiculus distinctly longer than scape, pedicel moderately elongated, ~ 2.5× as long as wide at apex, not flattened dorsoventrally, ~ 0.72× as long as segments 2 and 3 combined and 1.4× as long as segment 2 (Fig. 27). Surface of the scape shiny with diffused microreticulation, covered with moderately long and sparse, white, decumbent to subdecumbent hairs. Mandibles rounded, with deep striae, surface shiny with a few long and short yellow setae, cutting edge in large majors without teeth or with serrulate edge. ***Mesosoma*.** Moderately long, 2.0–2.1× as long as wide. Promesonotum regularly convex in profile with pronotum not or very slightly bulging above mesonotal plate (Figs 22, 25, 26), pronotal sides regularly rounded (Fig. 21). Propodeum positioned lower than promesonotum, flat to very slightly convex anteriorly then angulate posteriorly, posterior slope from obliquely flat to slightly concave thus propodeal angle distinct, forming angulate to obtuse tubercle (Figs 22, 25, 26). Pronotum anteriorly with transverse rugae and diffusely microreticulate interspaces but shiny, dorsum mostly irregular or with remnants of rugae, sides with sharp semicircular striae, and distinctly microreticulate interspaces but shiny, but in some populations lateral rugae diffused. Elevated dorsal plate of mesonotum mostly without rugae, or posteriorly with short longitudinal rugae, interspaces with diffused microreticulation, shiny, mesopleura with sharp perpendicular rugae and strong microreticulation. Propodeum dorsally, on dorsal half of sides and on posterior face with sharp transverse rugae, in metapleural area with sharp longitudinal rugae, interspaces anteriorly with diffused microreticulation, on metapleuron and posterior face of propodeum smooth and shiny. In specimens with predominantly red mesosoma rugae on propodeum less sharp than in dark specimens. Vestiture and setation of mesosoma sparse, pronotum with 6–8 long, yellow erect setae, the longest with length 0.290, sides of the pronotum without suberect setae, mesonotum anteriorly with 10–12 and posteriorly 4–6 long erect setae, usually also with a pair of long setae close to margin of propodeum, metapleuron in posterolateral corners with short subdecumbent setae, propodeum with two or three pairs of long erect setae often broken in mature specimens. ***Petiole*.** Elongate, with long pedicel and moderately high triangular node, thin, PI ~ 1.5, anterior slope flat or only slightly concave, pedicel, and base of node with distinct reticulate sculpture, anterior face of node with diffused microreticulation, sides of node microreticulate and with two or three longitudinal rugae, posterior face of node microreticulate, without striation. Top of petiole angular, upper margin and sides with two or three erect setae on each side. ***Postpetiole*.** Rounded in profile, globular in dorsal view, 1.3–1.4× as wide as the petiole, surface microreticulate and posterolaterally with few fain transverse striae, whole surface with 6–8 long erect setae. ***Gaster*.** Whole surface of first tergite with extremely short and sparse appressed hairs and strongly diffused background microreticulation, in anterior third appearing more micropunctate than microreticulate, in posterior 2/3 length hardly visible or also reduced only to micropunctate, completely without erect setae except row of posterior submarginal setae; second tergite with two rows of long erect setae. ***Legs*.** Moderately elongate, femora distinctly swollen centrally, tibiae moderately widened apically, mid and hind tarsi longer than tibiae. Whole surface of femora diffusely microreticulated, dorsally and laterally covered with moderately sparse and long, decumbent and subdecumbent and ventrally semierect to erect setae. Surface of tibiae covered with sparse and long decumbent to semierect setae.

**Figures 23, 24.**
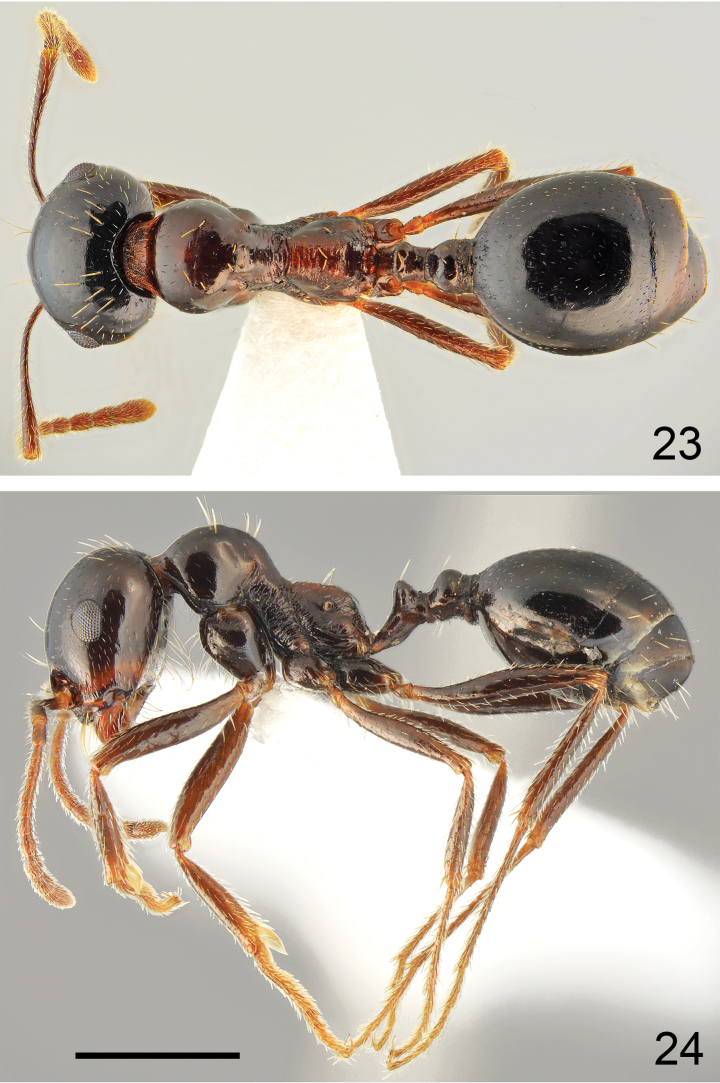
Minor worker of *Messorkardamenae* Salata & Borowiec, sp. nov., population from Kos **23** dorsal **24** lateral. Scale bar: 1 mm.

**Figures 25, 26.**
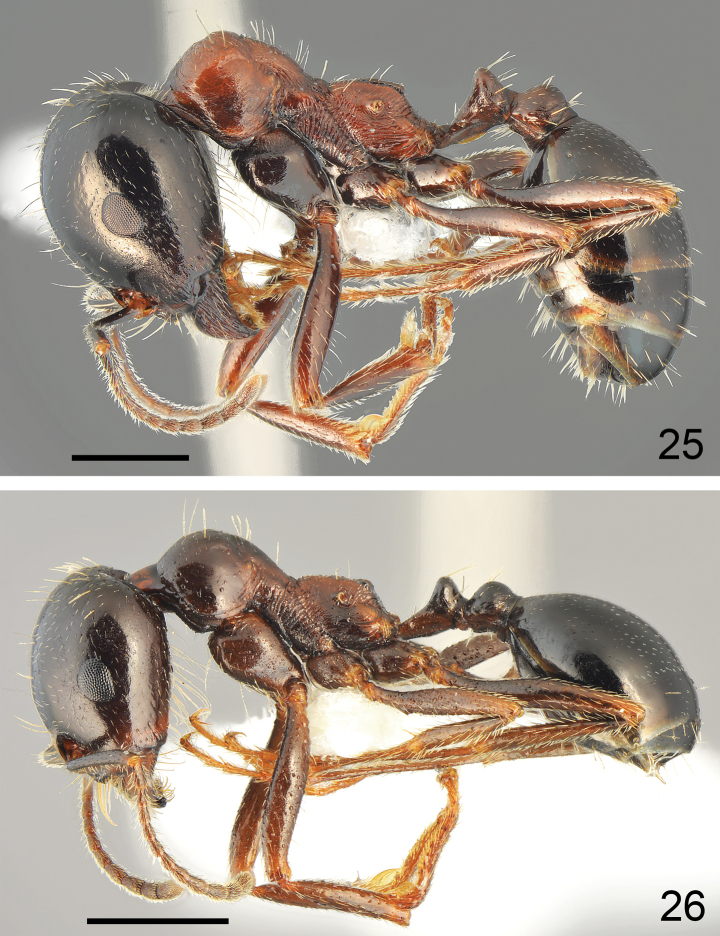
Worker of *Messorkardamenae* Salata & Borowiec, sp. nov. **25** major worker from Rhodes **26** medium worker from Nisyros. Scale bar: 1 mm.

**Figures 27, 28.**
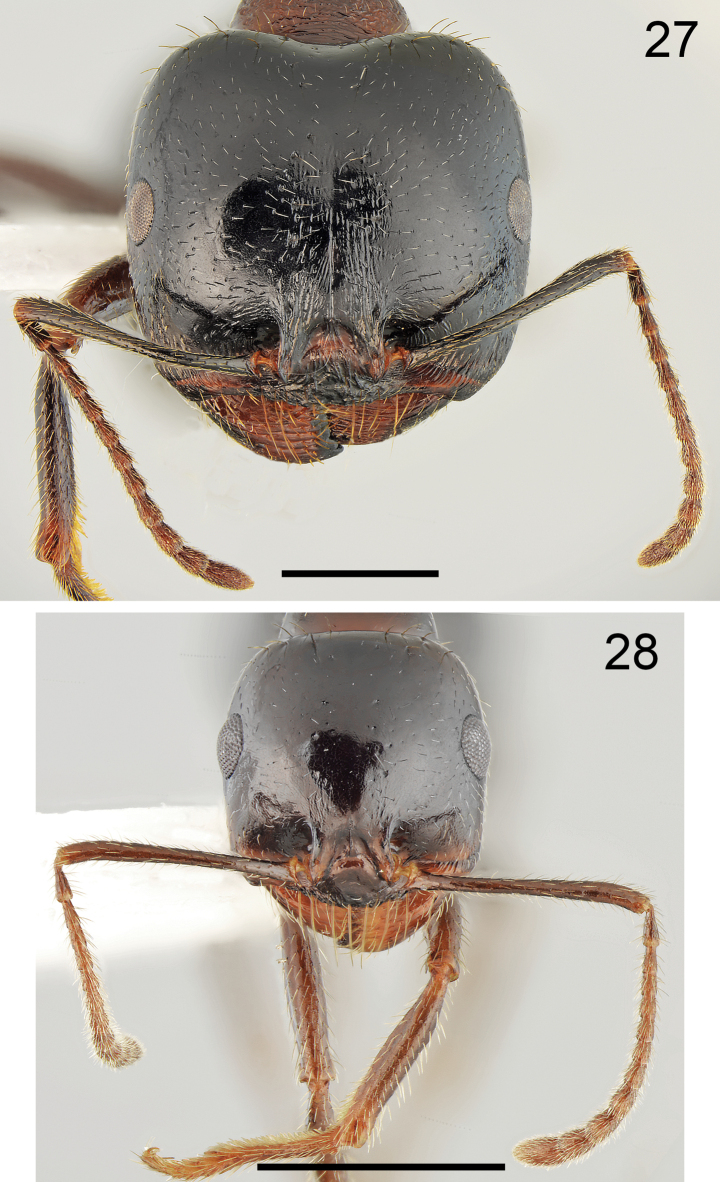
Head of *Messorkardamenae* Salata & Borowiec, sp. nov., population from Kos **27** major worker **28** minor worker. Scale bar: 1 mm.

Minor workers (*n* = 5): HL: 1.119–1.492 (mean 1.361); HW: 1.025–1.416 (mean 1.279); SL: 0.984–1.238 (mean 1.144); EL: 0.270–0.349 (mean 0.315); WL: 1.476–1.980 (mean 1.799); MW: 0.695–0.890 (mean 0.823); PSL: 0.156–0.190 (mean 0.179); PW: 0.241–0.314 (mean 0.282); PPW: 0.333–0.397 (mean 0.544); HL/HW: 1.042–1.099 (mean 1.067); SL/HW: 0.866–0.960 (mean 0.898); WL/MW: 2.124–2.227 (mean 2.183); EL/HL: 0.217–0.241 (mean 0.232); PSL/HW: 0.121–0.166 (mean 0.142); PPW/PW: 1.260–1.382 (mean 1.318).

***Colour*.** As coloured as major workers, but sometimes gena reddish, or head and mesosoma mostly reddish brown to brown (Figs 24, 26). ***Head*.** Slightly more elongated and more rounded in frontal view than in major workers, ~ 1.01× as long as wide, softly converging anterad and posterad, behind eyes more regularly rounded, occipital margin of the head slightly convex (Fig. 28). Clypeus in the middle completely smooth and shiny. Frons mostly smooth and shiny, with or without only few very short striae laterally, gena with shorter rugae than in majors. Background microreticulation of head almost visible or absent. ***Mesosoma*.** As slim as in majors, WL/MW ratio 2.1–2.2. Pronotal surface completely without rugae only with diffused microreticulation and micropunctation. Sculpture of mesonotum and propodeum finer than in majors, on sides of propodeum often mostly reduced to only strong microreticulation. Propodeal angle less marked than in majors with posterior face usually flat. Setation and vestiture of mesosoma as in majors but with lower number of setae, mesonotum with only 4–6 erect setae, propodeum with only one pair of setae or often without standing setae (Fig. 24). ***Petiole and postpetiole*.** As in major workers but surface with partly diffused reticulation and without striae and with smaller number of erect setae. ***Gaster*.** As shiny as in majors, with strongly diffused microreticulation, second tergite with only single transverse row of setae. Rest of characters as in major workers.

##### Comparative remarks.

*Messorkardamenae* sp. nov. with mean HW of the largest workers 2.63 (max 2.85) and HL 2.41 (max 2.55) is intermediate between large members of the *Messorwasmanni* complex (with mean HW of the largest workers > 2.65 (max 2.95) and HL > 2.46 (max 2.68)), and small members of the *Messorsemirufus* complex (with mean HW of the largest workers always < 2.00 and HL of the largest workers always < 1.95 (max 2.30 and 2.13 respectively)). *Messorkardamenae* sp. nov. clearly differs from *M.danaes* sp. nov. and *M.veneris* sp. nov. and both species of *M.wasmanni* complex: *M.concolor* Santschi and *M.wasmanni* Krausse in high numbers of setae in the occipital part of the head, usually 7–11 (up to 13) while other species have only 1–6 (up to 8) setae in the occipital area. *Messoratanassovii* with 12–20 large setae in occipital area looks the most similar. Both species have the same range of colour variation with mesosoma from uniformly red to almost completely brown to mostly black. *Messorkardamenae* sp. nov. is distinctly larger with ML of the largest workers 2.70–3.25 (mean 2.923) and HW 2.50–2.85 (mean 2.633) while in *M.atanassovii* ML is 2.32–2.73 (mean 2.395) and HW 1.86–2.30 (mean 1.980). Also, *M.kardamenae* sp. nov. has relatively shorter antennal scapes in major workers with SL/HW 0.64–0.68 (mean 0.659) while in *M.atanassovii*SL/HW is 0.68–0.74 (mean 0.721). In *M.kardamenae* sp. nov. the first gastral tergite is always lacking erect setae while in most populations of *M.atanassovii* the first gastral tergite is covered with very short and sparse erect setae, mostly limited to its basal third but sometimes setae are present also in posterior half of its surface. Both species are separated geographically, *M.kardamenae* sp. nov. is a southern species and occurs only in the eastern Dodecanese islands while *M.atanassovii* is a northern species noted from Bulgaria and north and western Greek provinces (Epirus, Ionian Islands, Eastern Macedonia, Thraki, and Central Macedonia). *Messorcreticus* differs in the presence of erect setae on the first gastral tergite and strong and never diffusing sculpture on mesosoma. From other species of the *M.semirufus* complex known from the eastern part of the Mediterranean basin, only similarly sized and coloured specimens of *M.intermedius* are similar but differ in the occipital part of the head entirely lacking erect setae or with at most two erect setae.

##### Biological notes.

Recorded from lowland habitats from sea level to an altitude 226 m. Observed in agricultural area, clay wasteland, ruderal places in tourist resort and on the soil in an active volcano.

##### Etymology.

Named after Kardamena, a woman whose name is the origin of the name of the type locality for *Messorkardamenae.* Kardamena is now a small town situated mid-way along the south coast of the island of Kos.

#### ﻿ Messor veneris

Taxon classificationAnimaliaHymenopteraFormicidae

Salata, Georgiadis & Borowiec sp. nov.

21A458AB-9BB3-5DBE-9A79-E19AB6F17D1B

https://zoobank.org/D87024EF-87BB-49FE-BA75-9C93C07E74B3

**Figures 29, 30.**
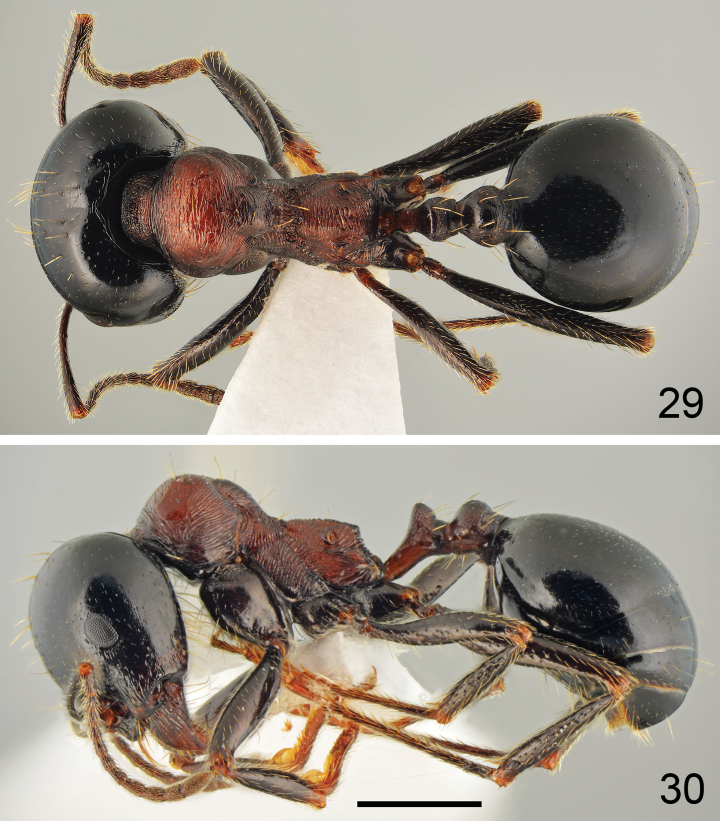
Major worker of *Messorveneris* Salata, Georgiadis & Borowiec, sp. nov., holotype **29** dorsal **30** lateral. Scale bar: 1 mm.

**Figures 31, 32.**
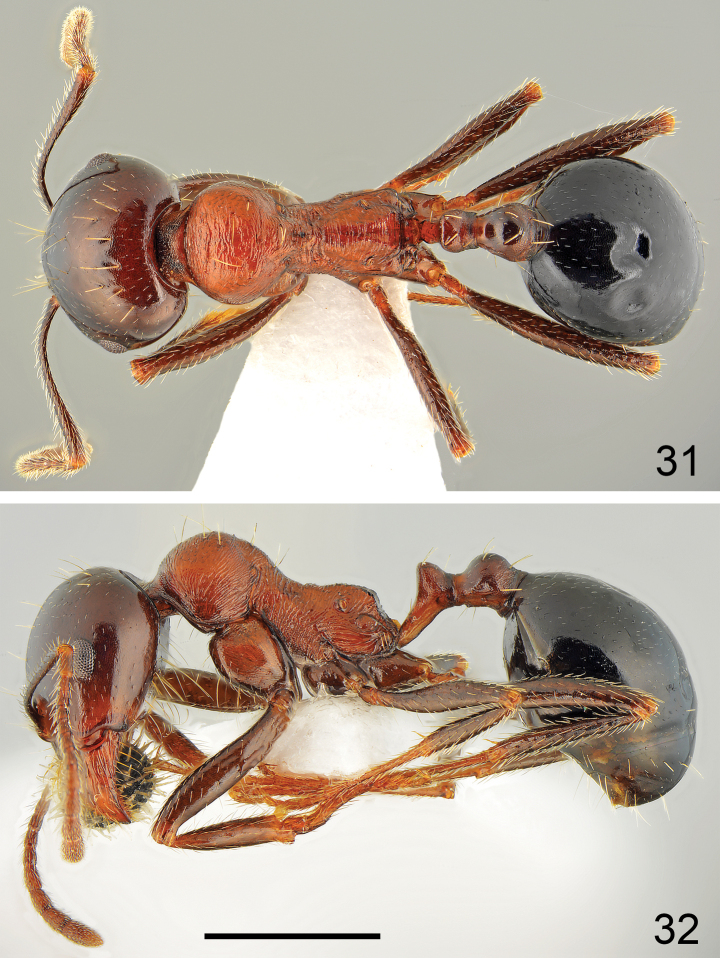
Minor worker of *Messorveneris* Salata, Georgiadis & Borowiec, sp. nov. **31** dorsal **32** lateral. Scale bar: 1 mm.

**Figures 33, 34.**
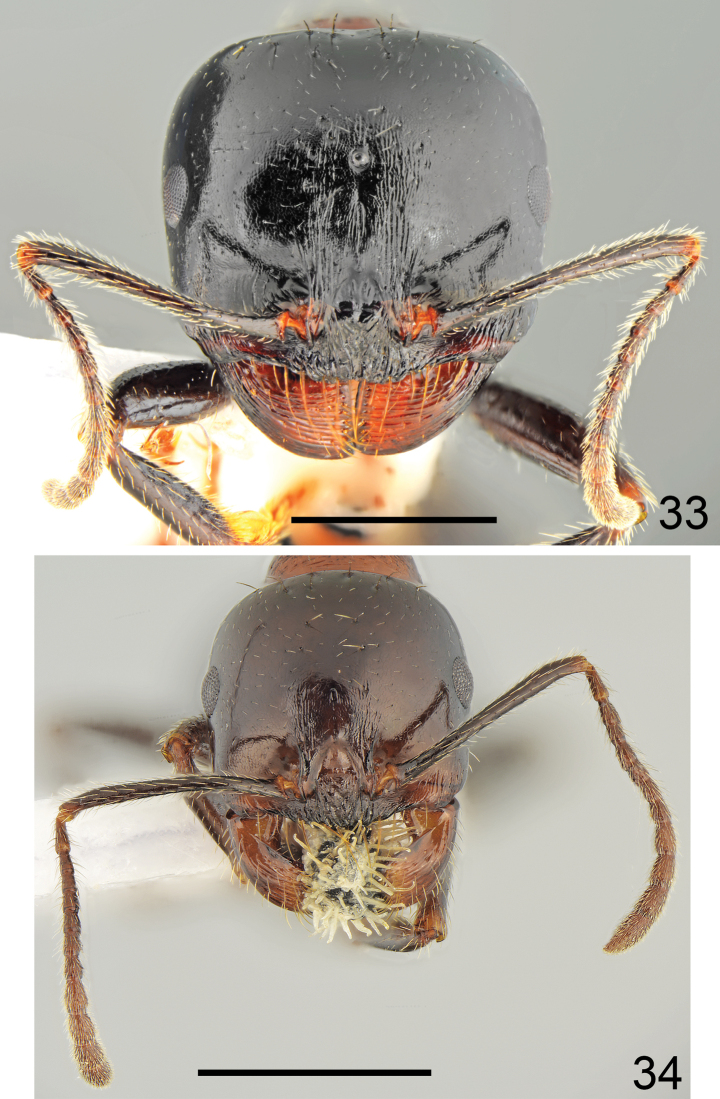
Head of *Messorveneris* Salata, Georgiadis & Borowiec, sp. nov. **33** major worker **34** minor worker. Scale bar: 1 mm.

Figs 29–30 , 31, 32 , 33–34

##### Type material.

***Holotype*** major worker (pin): GREECE, Cyclades, Milos, | Mount Elias loc. 2, 380 m | 36.6771, 24.3918, 05-06-| 2022 S. Salata CYC118 || Collection MNHW | Formicidae | MNHW-GR03205 (MNHW). 10 ***Paratypes***: 2 major, 6 medium, 3 minor workers (pin): the same data as for holotype (MNHW). 12 Paratypes: 7 major, 1 medium, 4 minor workers: the same data as holotype but different nest “Collection MNHW | Formicidae | MNHW-GR03204” (MNHW); 10 paratypes: 5 major, 1 medium, 4 minor workers: GREECE, Cyclades, Milos, | Mount Elias loc. 1, 369 m | 36.6783, 24.3898, 05-06-| 2022 S. Salata CYC117 || Collection MNHW | Formicidae | MNHW-GR03203 (MNHW).

##### Other material examined.

Greece • 4w. (pin), 18w. (EtOH); the same data as holotype (MNHW). • 22w. (EtOH); Cyclades, Milos, Mount Elias loc. 1; 36.6783, 24.3898; 369 m; 05 Jun 2022; leg. S. Salata (MNHW).

##### Description.

***Measurements*.** Major workers (*n* = 5): HL: 1.740–1.920 (mean 1.813); HW: 1.764–2.044 (mean 1.890); SL: 1.273–1.429 (mean 1.353); EL: 0.317–0.360 (mean 0.331); WL: 2.067–2.367 (mean 2.227); MW: 1.016–1.151 (mean 1.080); PSL: 0.241–0.302 (mean 0.267); PW: 0.349–0.413 (mean 0.379); PPW: 0.476–0.548 (mean 0.514); HL/HW: 0.929–0.977 (mean 0.960); SL/HW: 0.699–0.741 (mean 0.722); WL/MW: 2.034–2.083 (mean 2.062); EL/HL: 0.172–0.191 (mean 0.182); PSL/HW: 0.132–0.150 (mean 0.141); PPW/PW: 1.327–1.396 (mean 1.357).

***Colour*.** Head mostly black, only mandibles reddish to reddish brown and frontal triangle reddish, often anterior margin of gena also reddish. Occasionally whole gena reddish and ventral side of head reddish brown. Mesosoma usually red with obscure anterior margin of pronotum and ventral margin of mesonotum and propodeum. Petiole and postpetiole reddish, gaster black, only tergites with reddish or yellowish transparent hind margins. Coxa reddish brown to brownish black, femora and tibiae brown to black, basal segments of hind and mid tarsi brown, apical segments of hind and mid tarsi and whole fore tarsi yellowish to reddish. Antennal scapes mostly dark brown to black with yellowish brown apex, funicle usually completely brown or dark brown basally and yellowish brown apically (Figs 29, 30). ***Head*.** Subrectangular, 0.93–0.98× as long as wide, sides below eyes slightly converging anterad, above eyes subparallel then softly convex, posterior margin shallowly concave (Fig. 33). Anterior clypeal margin straight, without or with very shallow median emargination, with a row of ten long marginal, yellowish setae, the longest as long as length of clypeus. Clypeus without appressed pubescence, laterally with single erect seta and long erect seta close to the middle of lateral margin of central plate. Surface of the clypeus irregular, with short longitudinal to slightly irregular rugae, median clypeal carinae absent, interspaces microreticulate but shiny (Fig. 33). Eyes small and broadly oval, 0.5–0.6× as long as the gena. Frontal triangle impressed, with smooth surface and 2–4 short longitudinal striae, shiny. Frontal carinae short, slightly extending beyond frontal lobes. Frons narrow, in the narrowest part ~ 0.25× as wide as head width. Antennal fossa deep, surrounded by semicircular striae, surface smooth or with diffused microreticulation, shiny. Head mostly with regular and clearly marked background microreticulation and extremely small and sparse pits but shiny. Frons on almost whole surface with clearly marked longitudinal striae and with very narrow short shallow median sulcus, usually with large ocellar cavity behind the median sulcus, gena with short longitudinal striae, area behind eyes as microreticulate as rest of head, without striation. Surface covered with extremely short and sparse, hardly visible white appressed pubescence. Frontal lobes with single long erect seta, and frons behind frontal carinae with single long erect seta and laterally to ocellar cavity with two irregular rows of three or four setae, often broken in mature specimens. Vertex with 6–8 long erect setae placed closer to median impression than in occipital corners, which are without or with single short semierect setae, sides of the head and gena without standing setae, occasionally gena with one or two short semierect setae. Ventral part of the head and inner margin of mandibles with numerous long erect setae, partly forming a J-shaped psammophore. Antennal scape short, in frontal view almost straight only apically slightly curved, without preapical constriction, 0.70–0.74× as long as the width of the head; base of scapus moderately extended, outer angle acute, inner angle angulate. Funiculus distinctly longer than scape, pedicel moderately elongated, ~ 2.5× as long as wide at apex, not flattened dorsoventrally, ~ 0.68× as long as segments 2 and 3 combined and 1.4× as long as segment 2 (Fig. 33). Surface of the scape with diffused microreticulation, shiny, covered with long and sparse, white, subdecumbent to suberect hairs. Mandibles rounded, with deep striae, surface shiny with a few long and short yellow setae, cutting edge in large majors without teeth or with serrulate edge. ***Mesosoma*.** Moderately long, 2.0–2.1× as long as wide. Promesonotum regularly convex in profile with pronotum not or very slightly bulging above mesonotal plate (Fig. 30), pronotal sides regularly rounded (Fig. 29). Propodeum positioned lower than promesonotum, flat to very slightly convex anteriorly then angulate posteriorly, posterior slope distinctly concave; thus propodeal angle distinct, forming angulate tubercle (Fig. 30). Pronotum anteriorly and dorsally with transverse rugae and diffusely microreticulate interspaces but shiny, often anterolateral corners of pronotum with diffused rugae, sides of pronotum with distinct oblique to semicircular striae, and distinctly microreticulate interspaces but shiny. Elevated dorsal plate of mesonotum mostly with transverse rugae, sometimes diffused on top of dorsal plate, interspaces with diffused microreticulation, smooth and shiny, mesopleura with sharp perpendicular rugae and strong microreticulation. Propodeum dorsally, on dorsal half of sides and on posterior face with sharp transverse rugae, in metapleural area with sharp longitudinal rugae, interspaces anteriorly with diffused microreticulation, on metapleuron and posterior face of propodeum smooth and shiny. Vestiture and setation of mesosoma sparse, pronotum with 4–6 long, yellow erect setae, the longest with length 0.300, sides of the pronotum without suberect setae, mesonotum anteriorly and posteriorly with four long erect setae, metapleuron in posterolateral corners with short subdecumbent setae, propodeum with one pair of long erect setae often broken in mature specimens. ***Petiole*.** Elongate, with long pedicel and moderately high triangular node, thin, PI 1.5–1.6, pedicel, and base of node with distinct reticulate sculpture, anterior face of node smooth and shiny, sides of node microreticulate and posterior face of node microreticulate and often with fine transverse striation. Top of petiole angulate; upper margin and sides with eight erect setae. ***Postpetiole*.** Rounded in profile, globular in dorsal view, 1.3–1.4× as wide as the petiole, whole surface with 8–10 long erect setae and few suberect hairs on top. ***Gaster*.** Whole surface of first gastral tergite with well-marked, regular background microreticulation, surface slightly shagreened but shiny, covered with extremely sparse and short appressed hairs, completely without erect setae; second gastral tergite without long erect setae but usually with two short setae close to basal margin. ***Legs*.** Moderately elongate, femora distinctly swollen centrally, tibiae moderately widened apically, mid and hind tarsi longer than tibiae. Whole surface of femora diffusely microreticulated, dorsally and laterally covered with moderately sparse and long, decumbent and subdecumbent and ventrally semierect to erect setae. Surface of tibiae covered with sparse and long decumbent to semierect setae.

Minor workers (*n* = 5): HL: 1.063–1.190 (mean 1.120); HW: 1.032–1.159 (mean 1.093); SL: 0.937–1.056 (mean 0.982); EL: 0.206–0.238 (mean 0.222); WL: 1.436–1.611 (mean 1.514); MW: 0.675–0.762 (mean 0.716); PSL: 0.179–0.209 (mean 0.192); PW: 0.232–0.270 (mean 0.252); PPW: 0.302–0.349 (mean 0.333); HL/HW: 1.007–1.044 (mean 1.052); SL/HW: 0.878–0.911 (mean 0.899); WL/MW: 2.064–2.187 (mean 2.117); EL/HL: 0.191–0.206 (mean 0.199); PSL/HW: 0.167–0.184 (mean 0.176); PPW/PW: 1.269–1.366 (mean 1.321).

***Colour*.** Coloured as major workers, but sometimes vertex of head reddish and sides of head reddish brown (Figs 31, 32). ***Head*.** Slightly more elongated and more rounded in frontal view than in major workers, 1.01–1.04× as long as wide, softly converging anterad and posterad, behind eyes more regularly rounded, occipital margin of the head slightly convex (Fig. 34). Clypeus shiny, as sculptured as in majors. Frons mostly smooth and shiny, with short striae laterally, gena with shorter rugae than in majors. Background microreticulation of head not as distinct as in majors but visible on whole head surface. ***Mesosoma*.** Almost as slim as in majors, WL/MW ratio ~ 2.1. Pronotal surface with finer rugae than in majors. Sculpture of mesonotum and propodeum as in majors. Propodeal angle less marked than in majors but posterior face of propodeum shallowly concave. Setation and vestiture of mesosoma as in majors but with lower number of setae, mesonotum with only four erect setae, propodeum often without standing setae (Fig. 32). ***Petiole and postpetiole*.** As in major workers but surface with partly reduced reticulation and striae and with smaller numbers of erect setae. ***Gaster*.** Shinier than in majors, with background microreticulation slightly diffused, especially in posterior half of first gastral tergite but always visible on whole surface. Rest of characters as in major workers.

##### Comparative remarks.

*Messorveneris* sp. nov. with *M.danaes* sp. nov. are the smallest Balkan members of the *M.semirufus* complex. The largest majors of *M.veneris* sp. nov. have HW and HL < 2.0 mm (only two of the studied specimens of *M.veneris* sp. nov. have HW > 2.0 mm but < 2.1 mm and all studied specimens of *M.danaes* sp. nov. have HW < 2.0 mm). They are clearly characterised by low numbers of occipital setae, always less than nine. *Messorveneris* sp. nov. clearly differs from *M.danaes* sp. nov. in bicoloured body with mesosoma completely or predominantly red while in *M.danaes* sp. nov. the mesosoma is black. *Messorveneris* sp. nov. has very distinct and regular background microreticulation of head and first gastral tergite while in *M.danaes* sp. nov. the head and gaster are smooth and shiny, only with diffused and, especially on the head, hardly visible background microreticulation. Forms of *M.atanassovii*, *M.creticus* and *M.kardamenae* sp. nov. with predominantly red mesosoma clearly differ in numerous occipital setae (7–20 vs 6–8 in *M.veneris* sp. nov.). *Messorkardamenae* sp. nov. also differs in relatively shorter antennal scapes with SL/HW ratio 0.64–0.68 (mean 0.659) vs 0.70–0.74 (mean 0.722) in *M.veneris* sp. nov. From other species of the *M.semirufus* complex known from the eastern part of the Mediterranean basin, only *M.syriacus* is similar but differs in having larger body with HW in the largest majors up to 2.3 mm and in less evident head sculpture with strongly diffused background microreticulation and the posterior face of propodeum obliquely flat, and never concave.

##### Etymology.

Named after Venus de Milo (= Aphrodite of Milos), an ancient Greek sculpture discovered by a Greek farmer named inside a buried niche within the ancient city ruins of Milos – the terra typica of *Messorveneris*. The epithet is genitive.

#### ﻿ Messor creticus

Taxon classificationAnimaliaHymenopteraFormicidae

Salata & Borowiec, 2019

C9A32F81-8226-51AA-B30E-21BBC30ED98F

Messor creticus Salata & Borowiec, 2019: 58, figs 9–12 (s.w.); Greece (Crete I.).

##### Type material.

***Holotype*** worker: Greece: W Crete I., 1034, Omalos Plateau, 35°20'N, 23°53'E, 3.v.2011, LBC-GR00505, coll. L. Borowiec. ***Paratype*** material: 22 paratype workers, 1 paratype queen: same as for holotype (MNHW, NHMC).

##### Other material.

Greece • 1w. (EtOH); Crete, Chania Prov., Gramvousa peninsula; 35.56667, 23.58333; 80 m; 12 Jul 1997; leg. P. Lymberakis (NHMC). • 1w. (EtOH); Crete, Chania Prov., Korfos or Kefala; 34.83333, 24.1; 55 m; 14 Jun 1997; leg. K. Paragamian (NHMC). • 3w. (EtOH); Crete, Chania Prov., Omalos; 35.31667, 23.9; 1122 m; 05 May 2014; leg. S. Salata, (MNHW). • 4w. (EtOH); Crete, Heraklion Prov.: Rouvas Forest loc. 1; 35.15, 24.93333; 1316 m; 06 May 2014; leg. S. Salata (MNHW); • 4w. (EtOH); Crete, Heraklion Prov., Rouvas Forest loc. 2; 35.15, 24.83333; 1089 m; 28 Mar 2014; leg. S. Salata (MNHW). • 3w. (EtOH); Crete, Lasithi Prov.: above Kalimaki loc. 3; 35.11667, 25.43333; 1240 m; 25 Apr 2014; leg. S. Salata (MNHW).• 2w. (EtOH); Crete, Lasithi Prov., Chamaitoulo; 35.03333, 26.2; 180 m; 06 Aug 2000; leg. M. Chatzaki (NHMC). • 1w. (EtOH); Crete, Lasithi Prov., Chamaitoulo; 35.03333, 26.2; 180 m; 12 Oct 2000; leg. M. Chatzaki (NHMC). • 2w. (EtOH); Crete, Lasithi Prov., Chamaitoulo; 35.03333, 26.2; 180 m; 16 Mar 2001; leg. S. Simaiakis (NHMC). • 2w. (EtOH); Crete, Lasithi Prov., Dikti mt.; 35.11667, 25.46667; 1450 m; 05 Aug 2000; leg. S. Simaiakis (NHMC). • 1w. (EtOH); Crete, Lasithi Prov., Dikti mt.; 35.11667, 25.46667; 1450 m; 09 Jan 2001; leg. S. Simaiakis (NHMC). • 1w. (EtOH); Crete, Lasithi Prov., Milatos; 35.3, 25.58333; 310 m, 12 Jul 2000; leg. M. Chatzaki (NHMC). • 3w. (EtOH); Crete, Rethymno Prov., Nida plateau; 35.2, 24.83333; 1370 m; 01 V 2014; leg. S. Salata (MNHW). • 5w. (EtOH); Crete, Rethymno Prov., road to Nida plateau; 35.25, 24.88333; 1166 m; 25 Apr 2014; leg. S. Salata (MNHW). • 1w. (EtOH); Crete, Rethymno Prov., Afentis Christos; 35.23333, 24.7; 650 m; 21 Oct 1999; leg. E. Nikolakakis (NHMC). • 2w. (EtOH); Crete, Rethymno Prov., Moni Preveli; 35.15, 24.46667; 15 m; 26 Aug 1996; leg. M. Maroukli (NHMC).

##### Description.

See Salata and Borowiec (2019): 58.

##### Diagnosis.

*Messorcreticus* differs from *M.danaes* sp. nov., *M.veneris* sp. nov., and *M.kardamenae* sp. nov. in the presence of more erect setae on the occipital part of the head, presence of erect setae on the first gastral tergite, mesosoma entirely covered with thick sculpture and lack of smooth patches on its dorsal surface. The most similar is *M.atanassovii* but *M.creticus* differs in stronger sculpture on propodeum, which is entirely covered with thick and sparser rugae, and its dorsum does not bear reduced sculpture or smooth patches. Also, both species are separated geographically, *M.atanassovii* is a northern species noted from Bulgaria and north and western Greek provinces (Epirus, Ionian Islands, Eastern Macedonia, Thraki, and Central Macedonia) while *M.creticus* is a southern species and occurs only in Crete.

##### Distribution.

Greece: Cretan endemic species.

## Supplementary Material

XML Treatment for Messor atanassovii

XML Treatment for Messor danaes

XML Treatment for Messor kardamenae

XML Treatment for Messor veneris

XML Treatment for Messor creticus

## Footnotes

Note: An asterisk (*) is used to indicate those taxa that are included in the key below, which is restricted to taxa recorded from Greece.

## ﻿Additional information

### Author ORCIDs

Sebastian Salata https://orcid.org/0000-0003-0811-2309

Albena Lapeva-Gjonova https://orcid.org/0000-0003-0811-0768

Christos Georgiadis https://orcid.org/0000-0002-2728-3122

Lech Borowiec https://orcid.org/0000-0001-5668-6855

### Data availability

All of the data that support the findings of this study are available in the main text.
